# High-Throughput Analysis of Stimulus-Evoked Behaviors in *Drosophila* Larva Reveals Multiple Modality-Specific Escape Strategies

**DOI:** 10.1371/journal.pone.0071706

**Published:** 2013-08-20

**Authors:** Tomoko Ohyama, Tihana Jovanic, Gennady Denisov, Tam C. Dang, Dominik Hoffmann, Rex A. Kerr, Marta Zlatic

**Affiliations:** Janelia Farm Research Campus, Howard Hughes Medical Institute, Ashburn, Virginia, United States of America; Universitaet Regensburg, Germany

## Abstract

All organisms react to noxious and mechanical stimuli but we still lack a complete understanding of cellular and molecular mechanisms by which somatosensory information is transformed into appropriate motor outputs. The small number of neurons and excellent genetic tools make *Drosophila* larva an especially tractable model system in which to address this problem. We developed high throughput assays with which we can simultaneously expose more than 1,000 larvae per man-hour to precisely timed noxious heat, vibration, air current, or optogenetic stimuli. Using this hardware in combination with custom software we characterized larval reactions to somatosensory stimuli in far greater detail than possible previously. Each stimulus evoked a distinctive escape strategy that consisted of multiple actions. The escape strategy was context-dependent. Using our system we confirmed that the nociceptive class IV multidendritic neurons were involved in the reactions to noxious heat. Chordotonal (ch) neurons were necessary for normal modulation of head casting, crawling and hunching, in response to mechanical stimuli. Consistent with this we observed increases in calcium transients in response to vibration in ch neurons. Optogenetic activation of ch neurons was sufficient to evoke head casting and crawling. These studies significantly increase our understanding of the functional roles of larval ch neurons. More generally, our system and the detailed description of wild type reactions to somatosensory stimuli provide a basis for systematic identification of neurons and genes underlying these behaviors.

## Introduction

Understanding sensory-motor transformations at the level of genes, neurons and circuits has important implications for neurobiology and medicine. The somatosensory system of *Drosophila* larva is an especially tractable model system for tackling this problem, due to the small number of neurons (ca. 10,000) in its nervous system and excellent genetic tools for selective manipulation of single neuron types [1], [2]. Furthermore, the organization of somatosensory afferents and motor neuron dendrites in the larval ventral nerve cord not only resembles their organization in adult flies and other insects [3], [4], but also the organization of the vertebrate spinal cord [5], [6].

*Drosophila* larvae respond to somatosensory stimuli with stereotyped behaviors. In the absence of stimuli, larvae generally engage in rhythmic peristaltic crawling interrupted by exploratory head casting [7], [8]. Noxious mechanical and thermal stimuli can evoke sideways rolling, a stereotyped escape response [9]. Two mechanical stimuli, touching and vibration, induce head retraction and head casting [10]–[12]. Studies using targeted silencing of distinct classes of somatosensory neurons have identified nociceptive [13], mechanosensory [11], [14] and proprioceptive neurons [15]–[17]. The nociceptive sensory neurons also mediate, in part, larval avoidance of strong light [18]. Several ion channels essential for mechanical and thermal nociception and numerous other genes involved in the function and development of somatosensory neurons have been identified over the years [9], [19]–[22].

In principle, the excellent genetic tools available in *Drosophila* could allow systematic identification of all neurons and genes involved in somatosensation and somatosensory-guided behaviors [2], [23]. However, high-throughput screens have been difficult due to the low throughput of the single animal behavioral assays [9], [10], [13], [14] or by the inability to quantify larval reactions to somatosensory stimuli, such as rolls, in an automated way [24]. Recently high throughput methods have been developed for studies of larval chemotaxis [25], but they were lacking for studies of somatosensory-evoked behaviors.

**Figure 1:**
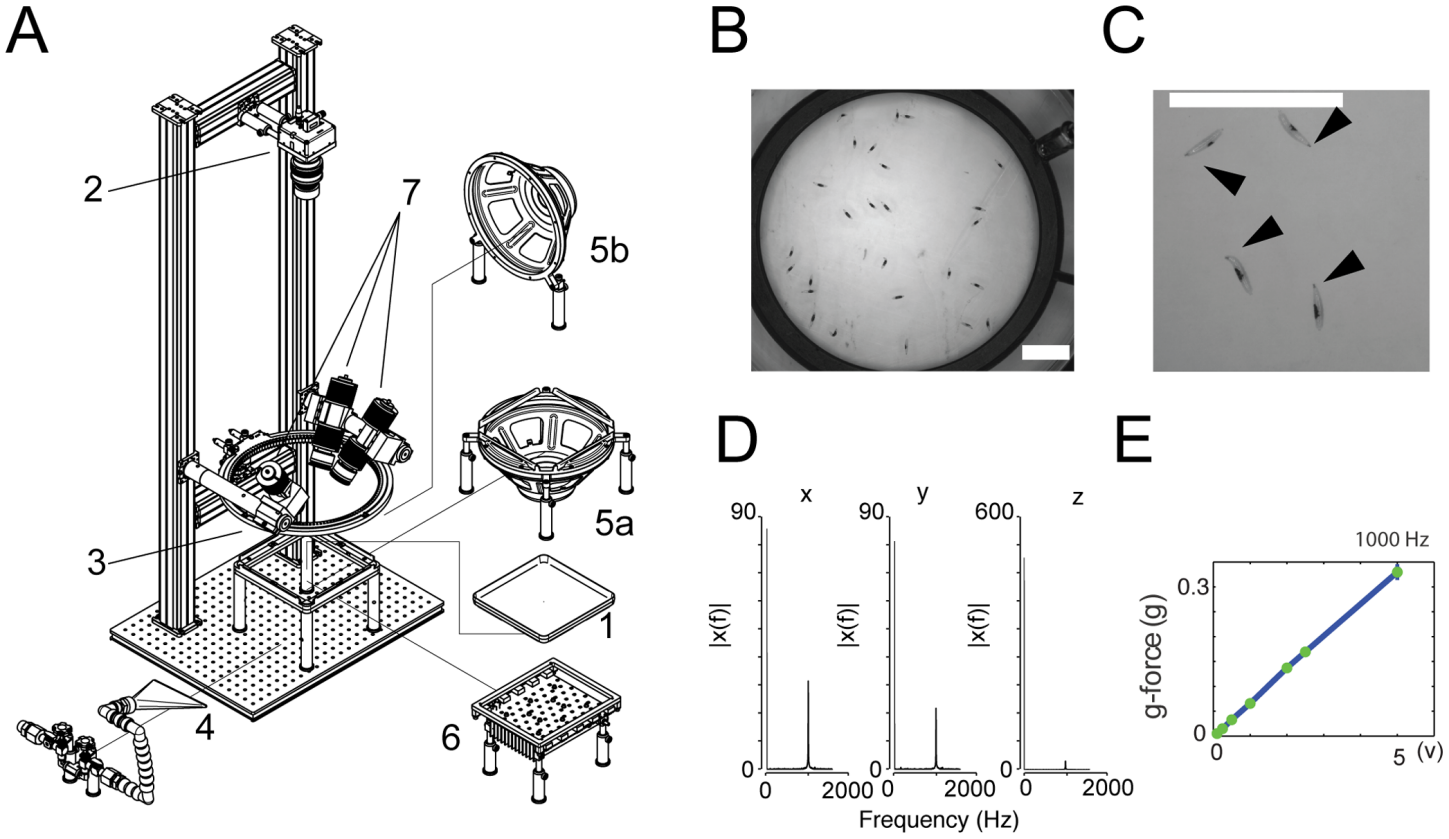
Hardware for somatosensory and optogenetic stimulation of *Drosophila* larvae. (A) Larvae roam a plastic dish filled with agar (1). A high-resolution camera (2) collects images to track their movement and body shapes. A ring light (3) provides illumination. Different hardware modules impart stimuli: air current through a 3D-printed flare nozzle (4) connected to plant-supplied compressed air, vibration and sound through a speaker (5a or 5b), blue light for ChR2 activation through an array of high-power blue LEDs (470 nm) underneath the arena (6), and noxious heat through high-power IR light (808 nm) delivered from solid-state lasers (7). (B and C) Snapshots of animals at the start of the experiment on the nociceptive stimulation rig, showing dots for absorption of the 808 nm laser light. Scale bar = 5 cm. Average dot size was 73.28 mm^2^±4.32 mm^2^ (s.e.m.). Dots can be placed on the top (B) or on the side (C) to study the directionality of the response. Movie S1 shows larvae with dots on the top rolling in response to 808 nm laser stimulation. Larvae roll in random directions. Movie S2 shows larvae with dots on the left hand side rolling to the left. (D and E) Characterization of the vibration frequency and g-force on agar surface in our rig. (D) Spectral power density plots (|X(f)|) obtained with Fast Fourier Transform (FFT) analysis of the acceleration of the agar surface of the arena when the speaker played 1,000 Hz tones (2 V) (X, Y, Z axis). The spectrum at 1000 Hz is normalized by the spectrum at 0 Hz which represents gravity ( = 1 g). (E) G-force at 1000 Hz increases linearly with increasing voltage applied to the speaker.

**Figure 2:**
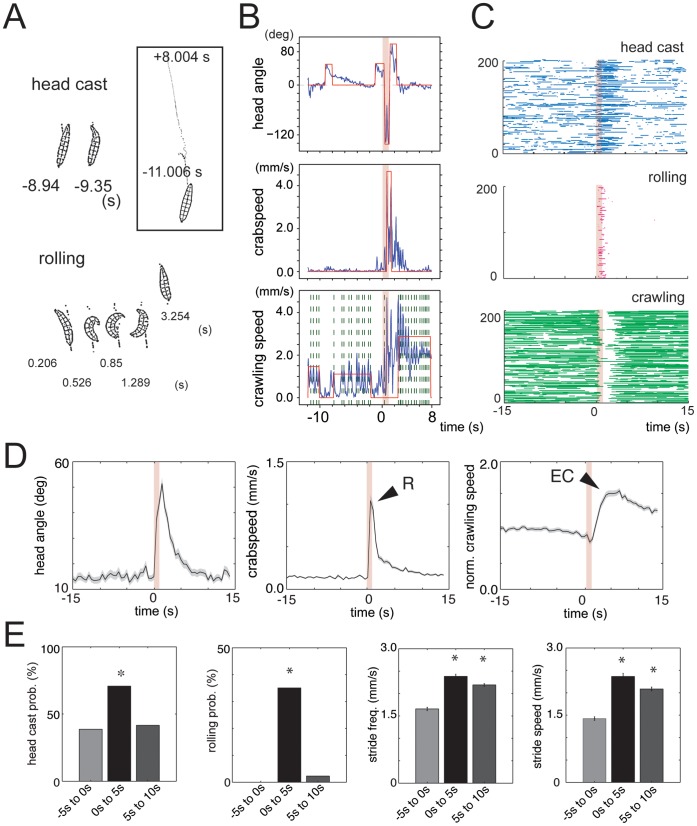
Noxious heat evokes *rolling* and *escape crawling*. (A) Stills from a representative tracking movie of contours of larvae obtained with the MWT software. Top: example stills before the noxious stimulus onset (0 s) during which larva was crawling straight (−8.94 s) and head casting (−9.35 s). Bottom: example stills after stimulus onset during which larva was rolling (0.526, 0.85, 1.289 s) and then crawling straight again (3.254 s). Box: the path (x and y positions of the center of mass) taken by the same larva from −11.006 s before stimulus to +8.004 s after stimulus. (B) Graphs show head angle (top), crabspeed (middle) and crawling speed (bottom) of the larva shown in (A) as a function of time (blue lines) and casts (top), rolls (middle) and crawling runs (bottom) automatically detected by LARA (red lines). 808 nm laser stimulation came on at 0 s (1 s duration). The red line is zero if no action is detected and non-zero whenever the action is detected. For head casts, upward and downward deflections indicate leftward and rightward casts, respectively. Vertical green dashed lines in the bottom panel mark maxima in the speed function, which correspond to individual peristaltic crawling strides, automatically detected by LARA. Note how 808 nm light stimulus evoked a peak in crabpseed function (middle) detected as a rolling event and then a crawling run (bottom panel) with greater stride frequency and stride speed than the runs prior to stimulation. (C) Behavior raster plots show periods during which an individual larva was head casting or bending (top), rolling (middle) or crawling (bottom) during a time interval from 15 s prior to 15 s following stimulation. 808 nm laser stimulation came on at 0 s (1 s duration). Each row represents one larva tracked continuously throughout the interval (a total of 200 animals). (D and E) Reactions of wild-type *Canton S* larvae to noxious heat. (D) Graphs show head angle, crabspeed and normalized crawling speed as a function of time averaged across many animals from experiments in which wild-type *Canton S* larvae were presented with noxious heat stimuli at ambient temperature of 32°C. Dark lines, mean value. Light lines, ± s.e.m. N = 154. Pink lines at 0 s mark the stimulus onset and duration. R, roll. EC, escape crawl. Normalized crawling speed was computed from the absolute crawling speed by dividing the absolute crawling speed at each time point after stimulation with the mean value of crawling speed of that animal before stimulation. Graphs highlight the dynamics of the reactions to noxious heat. Following noxious heat stimulation there is first a peak in the crabspeed function, which corresponds to the roll (R), followed by a peak in the mean normalized speed function, that corresponds to the escape crawl (EC). Crabspeed peaks at 1 sec following stimulation. The speed of escape crawl is 50% higher than that of the baseline crawl prior to stimulation and it peaks at ca. 4 sec following stimulation. (E) Bar charts show head casting and rolling probability and the mean value of the maximum stride speed and stride frequency in a 5 s time window before stimulation (−5 s to 0 s) and in two consecutive 5 s time windows after stimulation (0 s to 5 s and 5 s to 10 s). Error bars indicate s.e.m. * *p*<0.001, for window(s) after stimulation compared to window before stimulation. Rolling probability is 0 (N = 189 larvae) prior to stimulation, but is significantly increased to 35% in the 5 sec window following stimulation (*p*<10^−6^, N = 154 larvae). The increase in mean speed following stimulation shown in Fig. 2D is in part due to a significant increase in peristaltic crawling stride frequency (*p*<10^−6^, N = 167 peristaltic crawling runs) and peristaltic crawling stride speed (*p*<10^−6^, N = 167 peristaltic crawling runs) in the windows following stimulation, relative to the 5 sec window prior to stimulation.

In this paper, we present hardware modules that allow automated and temporally controlled stimulation of 30–100 freely crawling larvae at once with noxious heat, vibration, air current and/or optogenetic stimuli (Fig. 1), while recording videos of their behavior. We use custom signal processing software to extract in an automated way larval behavior raster plots from the video tracking data (software for LArval Reaction Analysis, LARA) (Fig. 2). While previous software available in the field for automated tracking of single larvae [26], or of populations of freely crawling larvae, allows automated quantification of peristaltic crawling runs and turns [25], [27], this is the first software that also allows quantification of hunches, rolls (key components of larval reactions to somatosensory stimuli) and individual peristaltic crawling strides in freely crawling larvae.

Using these methods we identified a number of novel behaviors. We showed for the first time that larvae react to air current. We found that each stimulus modality induced a characteristic escape strategy that consisted of multiple actions that occurred in a stereotyped sequence. Each stimulus modulated the probability, amplitude and timing of the actions in characteristic ways. Furthermore we found that the reactions to noxious heat and vibration were context-dependent and modulated by mild heat (32°C).

We confirmed that inactivation of the nociceptive, Class IV, neurons reduced the reactions to noxious heat, whereas inactivation of the mechanosensory, ch neurons reduced the reactions to mechanical stimuli. Consistent with this we showed for the first time calcium transients in a subset of ch neurons, in response to 1000 Hz vibration. We also showed for the first time that optogenetic activation of ch neurons increased the probability of head casting and increased crawling speed, reactions that were also evoked by vibration. The tools presented here and the detailed description of the wild type reactions to the somatosensory stimuli provide the basis for a systematic analysis of the function of neurons and genes in the integration of somatosensory information and in sensory-motor transformations.

## Materials and Methods

### Fly Stocks

We used GAL4-UAS system [28] to direct the expression of effector proteins to specific neuron subtypes. We used the following effector stocks: *w^+^;;UAS-Shibire^ts1^* [29], *w;UAS-ChR2*; *UAS-ChR2* [30] (double insertion stocks are gift from B. Condron lab) and *pJFRC12-10XUAS-IVS-myr::GFP* (Bloomington stock number: 32197; [23]). Throughout paper we used as wild-type controls the *Canton S* larvae, the progeny larvae from the *Canton S* stock, crossed to appropriate GAL4 lines, or the progeny larvae from the “empty” GAL4 vector insertion stock, *w;; pBDPGAL4U* [23] crossed to the appropriate effector. *w;; pBDPGAL4U* [23] were selected because they have the same genetic background as the 8,000 GAL4 lines recently made for inactivating or activating specific cell types in *Drosophila* [2], so that the wild-type behaviors we described could serve as controls for future neural inactivation and activation screens. We used the following specific sensory neuron driver lines: *w;;iav-GAL4* (gift of C. Montell, Johns Hopkins University) [31], *w;;R38A10* [1], *w;;R20C06* [1] (Li H-H., Kroll, J. R., Lennox, S., Ogundeyi, O., Jeter, J., Depasquale, G. and Truman, J., W., 2013, submitted). To characterize nociceptive stimuli, we used *painless^1^, painless^3^* [9] (gift of D. Tracey, Duke U) and *Dmpiezo* [19] (gift of A. Patapoutian).

### Larval Dissections and Immunocytochemistry

To analyze the expression pattern of the *R38A10-GAL4* and *R20C06-GAL4*, we crossed these lines to *pJFRC12-10XUAS-IVS-myr::GFP* (Bloomington stock number: 32197; [23]). 3^rd^ instar progeny larvae were placed in a phosphate buffered saline (PBS; pH 7.4) in Sylgard-coated dish, cut along the dorsal midline and the body wall pinned. Filleted larvae were fixed with 4.0% paraformaldehyde for 30 min at room temperature, and then rinsed several times in PBS with 0.4% Triton X-100 (PBS-TX). Primary antibodies were used at a concentration of 1∶1000 for rabbit anti-GFP (Invitrogen) and 1∶50 for mouse mAb 22C10 [32] and incubated overnight at 4°C. Secondary antibodies were anti-mouse Alexa563 (diluted 1∶250; Invitrogen) and anti-rabbit Alexa488 (diluted 1∶250; Invitrogen), respectively. After overnight incubation in secondary antibodies, the tissue was rinsed for several hours in PBS-TX, and mounted in PRoLong Gold Antifade (Invitrogen).

### GCaMP Imaging and Data Analysis

Third-instar foraging larvae were dissected in Schneider’s insect medium and before imaging replaced with HL-6 [33] that contains 2 mM Ca^2+^, 340 Osm, pH of 7.2, 7 mM L-glutamic acid added to reduce muscle movement. The larvae were dissected and pinned in a sylgard plate. Wide-field Ca^2+^ imaging was performed on an upright Olympus BX61-WI microscope using a 40× water immersion objective (0.8 NA), a 2×lens extender (EX2C), and an Andor EMCCD camera (Model DU897 BV, 512×512 pixels 30FPS, 100 EMgain 32.9 exposure; Andor Technology) with 10% illumination of X-CITE excite light source (Lumen Dynamics). The fluorescence filter set used was BrightLine 3035B (Semrock) (excitation, 472/30-25; emission, 520/35-25). We used a speaker (Dayton, ND90-8) mounted to the stage. The imaging system and the speaker were controlled by Methamorph. Data analysis was performed using MetaMorph software. Regions of interest (ROI) covering individual chrodotonal dendrites were selected for analysis. The images were pseudocolored using Fiji [34].

### Behavior Apparatus

The apparatus (Fig. 1A) comprises a video camera (DALSA Falcon 4M30 camera) for monitoring larvae, a ring light illuminator, a computer and the four hardware modules. The bill of materials (BOM), schematic diagrams and PCB CAM files (Gerber format) for the assembly of the apparatus will be available in Supplementary Material or at HHMI Janelia website.

Video microscopy was performed with red or yellow LED (Cree C503B-RCS-CW0Z0AA1 at 624 nm in the red or Cree C503B-ACN-VW0Y0341-0MT at 524 nm in the yellow) illumination (outside the range of larval phototaxis). Video was recorded at 15 frames s^−1^. For the noxious heat experiments the camera was mounted above a 9.3 cm diameter circular arena and a spatial resolution of up to 56 microns per pixels per larva. For the other experiments the camera was mounted above a 25×25 cm^2^ square resulting in a spatial resolution of 90 µm per pixel.

To study larval reactions to noxious thermal stimuli, light from three infrared solid-state multimode lasers (B&W Tek Model BWF5-808-75-HHMI) each outputting up to 75 W at 808 nm is delivered through a multi-mode fiber to a beam homogenizer (B&W Tek proprietary technology) ensuring a minimum of speckle variation (below 20%) in the spatial beam profile. The power output of the IR lasers is controlled directly through the lasers’ front-panel user interface. The light intensities at the arena were measured with a Thorlabs S314A thermal sensor hooked up to a Thorlabs PM100 optical power meter. In our experiments laser power was set to 65 W resulting in intensity of 40 mW/mm^2^ at the arena.

Air-current is delivered to a 25×25 cm^2^ arena at a pressure of 1.1 MPa through a 3D-printed flare nozzle with a 16 cm × 0.17 cm opening connected through a tubing system to plant-supplied compressed air (0.5 MPa converted to a maximum of 1.4 MPa using a Maxpro Technologies DLA 5-1 air amplifier, standard quality for medical air with dewpoint of −10°C at 90 psig; relative humidity at 25°C and 32°C, ca. 1.2% and 0.9%, respectively). The strength of the airflow is controlled through a regulator downstream from the air amplifier and turned on and off with a solenoid valve (Parker Skinner 71215SN2GN00). The nozzle is placed near the edge of the arena directing airflow at grazing incidence at it, carefully positioning it for even coverage of the plate. Air flow rates at different positions in the arena were measure with a hot-wire anemometer (Extech Model 407119A).

The module for presenting vibration consists of a speaker (120 W, 12 in diameter) and an amplifier (Pyle Pro PCA3) controlled by a signal generator (Tektronix AFG3021B). The speaker can be placed either below or next to the arena to generate vibrations between 25 Hz and 5 kHz, with an electronic signal of 1-V amplitude and 100 Hz generating a sound level of 122 dB at the assay plate (measured by a Realistic 33–2050 Sound Level Meter). The frequency of vibration of the agar surface was measured with an ADXL345 3-axis accelerometer assembled onto a small printed circuit board embedded in the agar. The power and communications were connected by flexible wires to a microprocessor and a computer via USB. The ADXL was configured for 3200 samples per s with a range of +/−2 g on each axis.

The optogenetic module consists of a circuit board that controls a 22 cm × 15.5 cm array of 60 LEDs placed below a large 25×25 cm^2^ arena spaced in such a way to cover the entire arena with light. For Channelrhodopsin activation we used a Philips Lumileds LUXEON Rebel emitting around 470 nm (LXML-PB01-0018). The intensity at the agar surface was measured using a Thorlabs S130A light sensor hooked up to a Thorlabs PM100 optical power meter. In the experiments we used 0.17 mW/mm^2^ produced by an LED drive current of 700 mA.

All our hardware modules are controlled through the MWT software http://sourceforge.net/projects/mwt [35]. The IR laser, the function generator, the air current relay and the LED circuit board are triggered through TTL pulses delivered by a Measurement Computing PCI-CTR05 5-channel, counter/timer board at the direction of the MWT. The onset and durations of all stimuli are also controlled through the MWT. All our rigs were inside 32.00″ wide × 28.00″ deep × 60.00″ high, temperature controlled enclosures, with temperature settable from ambient to 40°C in 0.1°C steps (Life Science Engineering, Inc.). The humidity in the room is monitored and held at 58%, with humidifiers (Humidifirst Mist Pac-5 Ultrasonic Humidifier).

### Behavior Experiments

Embryos were collected for 6–8 hours at 25°C or 14–16 hours at 18°C with 65% humidity. Larvae containing the *UAS-Shibire^ts1^* transgene were raised at 18°C for 6–7 days with normal cornmeal food. For the nociceptive behavior assay, wandering stage animals were used (7 day at 18°C). For the other experiments, foraging 3^rd^ instar larvae were used (6 day 18°C). Larvae for optogenetic activation experiments, containing the *UAS-ChR2* transgene, were grown in the dark at 25°C for 4 days on fly food containing trans-retinal (SIGMA R2500) at a final concentration of 500 µM. The no-retinal controls for these experiments were grown in the same way, only without retinal in the food.

Before experiments, larvae were separated from food using 15% sucrose, scooped with a paint brush into a sieve and washed with water. This is because sucrose is denser than water, and larvae quickly float up in sucrose making scooping them out from food a lot faster and easier. This method is especially useful for high-throughput screening. We have controlled for the effect and have seen no difference in the behavior between larvae scooped with sucrose and larvae scooped directly from the food plate with a forceps.

The larvae were dried and placed into the center of the arena. The substrate for behavioral experiments was 4% Bacto agar gel in a 25×25 cm^2^ square plastic dishes or a 9.3 cm diameter circular Petri dish (for nociceptive behavior assay). For experiments with *UAS-Shibire^ts1^* at restrictive temperature we incubated the larvae after washing in 32°C water for 2 min and then we placed them onto pre-warmed (32°C) agar-filled dishes. The dishes with larvae were placed on the rig inside the temperature-control enclosure, and the temperature was set to 32°C. For *UAS-Shibire^ts1^* experiments at permissive temperature, and for *UAS-ChR2,* larvae were washed with water at room temperature, the dishes were kept at room temperature and the temperature on the rig inside the enclosure was set to 25°C.

For the nociceptive behavior assay a black dot was painted on each larva by touching it quickly with a permanent marker (Sharpie, Rub a Dub), prior to placing the arena into the testing rig. We have confirmed that the dot itself without the 808 nm laser light did not alter significantly the baseline behavior of larvae compared to animals without dots. Snapshots of larvae were taken at the start of each experiment (Fig. 1B and 1C), allowing dot sizes to be quantified and dot positions to be monitored. Dots were segmented from the snapshots in an automated way in Fiji [34]. We have empirically determined the dot size range that works best and we try and always apply dots within that range. We have not observed a direct correlation between the dot size and the probability of rolling, other than at the two extremes. Very small dots (less than 10 mm^2^) did not evoke rolling, very big dots (more than 150 mm^2^) did not evoke rolling, likely due to extensive tissue damage. Average dot size in our experiments was 73.28 mm^2^±30.59 (standard deviation). Within the large range of dot sizes, rolling probability was constant for constant 808 nm laser light intensity. We observed rolling probability rise with the intensity of the 808 nm laser light at the arena, reaching a plateau at 40 mW/mm^2^. We therefore used this intensity for all our experiments. Dots can be placed on the top of the animal, towards the center of the dorsal midline (Fig. 1B) or on the side (Fig. 1C) to study the directionality of the response. All the experiments in this study, except movie S2, were done with dots on the top. We tested ca. 30 larvae at once for the nociceptive behavior assay and ca. 100 larvae at once for the other assays.

### Behavioral Analysis

#### General overview and computation of relevant variables

The LARA software package for detecting larval motor patterns from tracking data is available on http://sourceforge.net/projects/salam-hhmi. Larvae were tracked real-time using the MWT software http://sourceforge.net/projects/mwt [35]. We rejected objects that were tracked for less than 5 sec or moved less than one body length of the larva. For each larva MWT returns a contour, spine and center of mass as a function of time, as described in Swierczek et al 2011 [35]. Raw videos are never stored. From the MWT tracking data we computed the key parameters of larval motion, using specific Choreography (part of the MWT software package) variables that we tailored for larvae, as opposed to *C. elegans*.

Speed of the center of mass (speed) (mm/s) and sideways rolling speed (crabspeed) are computed as follows: (1) Given points on the animals path *p*(*t*), set *t*_a_ = *t* − *T*/2 and *t*_b_ = t+T/2, where *T* = 0.1 s is the integration time. (2) Increment *t*_a_ and decrement *t*_b_ while both still bracket *t* and while ||*p*(*t*_a_) − *p*(*t*_b_)|| increases, as a fast heuristic for calculating argmax_ta, tb_(||*p*(*t*_a_) − *p*(*t*_b_)||). (3) Let *b*(*t*) be a unit vector along the least-squares line fit to the segmented pixels that form the worm’s body. Then speed and rolling speed (crabspeed) are ||*p*(*t*_a_) − *p*(*t*_b_)|| and the magnitude of *p*(*t*_a_) − *p*(*t*_b_) perpendicular to *b*(*t*), respectively. Length (midline) (mm) is defined as the total Euclidean distance along the spine as in Swierczek et al. 2011 (where it is called “spine length”).

Width (mm) of the larva is defined as follows. Let *o*_L_(*i*) be the positions of the outline pixels on the left side of the thresholded image of the animal, and let *o*_R_(*i*) be those positions on the right. Let *n*_L_ and *n*_R_ be the number of outline pixels on each side, respectively. Let *ō*_L_(*i*) and *ō*_R_(*i*) be a 5-point boxcar average of o_L_(*i*) and *o*_R_(*i*) respectively. Then the morphological width associated with spine point *k* (0< = *k*<*m*; *m* = 11 typically) is the min_i,j_(||*ō*_L_(*i*) − *ō*_R_(*i*)||) where *n*_L_*(*k*−1)/*n*<*i*<*n*_L_*(*k*+1)/*n* and *n*_R_*(*k*−1)/*m*<*j*<*n*_R_*(*k*+1)/*m*. The overall morphological width is the mean of the widths of the central 60% of the spine points (typically 7 points out of 11). Head angle (cast) (deg) is cast is the signed distance from the least-squares line fit of the posterior 2/3 of the animal’s spine points (typically 7 points out of 11) to the point in the anterior 1/5 of the animal’s spine (typically 2 points out of 11) most distant from that line; sign is chosen to be positive to the right of the vector from tail to head along the least-squares line.

For further details of the software implementations of the above calculations see the open-source package http://sourceforge.net/projects/mwt. The exact Choreography commands that we used to obtain each of the variables for all animals from one run are as follows (see documentation on Choreography at http://sourceforge.net/projects/mwt, for definitions of all the parameters):

java -Xincgc -Xms8000m -Xmx8000m -jar/Users/Applications/Chore.jar -t 5 -s 0.1 -p 0.095 -M 1–shadowless –segment –nanless -o Dts1234 -O speed -N all.

java -Xincgc -Xms8000m -Xmx8000m -jar/Users/Applications/Chore.jar -t 5 -s 0.1 -p 0.095 -M 1–shadowless –segment –nanless -o Dtr1234 -O crabspeed -N all.

java -Xincgc -Xms8000m -Xmx8000m -jar/Users/Applications/Chore.jar -t 5 -s 0.1 -p 0.095 -M 1–shadowless –segment –nanless –plugin SpinesForward::rebias –plugin Reoutline::exp –plugin Respine::0.23::tapered = 0.28,1,2 -o Dtm1234 -O length -N all.

java -Xincgc -Xms8000m -Xmx8000m -jar/Users/Applications/Chore.jar -t 5 -s 0.1 -p 0.095 -M 1–shadowless –segment –nanless –plugin SpinesForward::rebias –plugin Reoutline::exp –plugin Respine::0.23::tapered = 0.28,1,2 -o DtM1234 -O width -N all.

java -Xincgc -Xms8000m -Xmx8000m -jar/Users/Applications/Chore.jar -t 5 -s 0.1 -p 0.095 -M 1–shadowless –segment –nanless –plugin Reoutline::exp –plugin Respine::0.23::tapered = 0.28,1,2–plugin SpinesForward::rebias –minimum-biased 3mm -o DtC1234 -O cast -N all.

Combinations of the functions of these variables are used by our newly developed LARA open-source software package to detect hunches, rolls and head casts using signal-processing algorithms, as described below. For details of the software implementation of the below described algorithms, see http://sourceforge.net/projects/salam-hhmi.

#### Automated detection of motor patterns and extraction of their features by LARA

Biologically meaningful actions are generally defined as significant “events” in one or more functions of variables extracted from MWT tracking data, stored as time series data and subsequently used as input by LARA. For the purposes of processing described in this paper, the following set of five input functions was used: speed of the center of mass (mm/s, Δt = 0.1); sideways rolling speed (crabspeed) (mm/s); spine length (mm); width of the larva (mm); head angle (deg).

Analysis of behavioral actions by LARA starts from detecting significant events in the individual functions used. An event is significant if it meets the criteria specified by the signal processing algorithms, as described below. In the simplest case, a behavioral action is simply an event in the “key” function corresponding to that action. For the crawling, rolling, casting and hunching actions, the key functions are, respectively, the speed, crabspeed, cast and midline. For non-oscillating signals, such as cast, crabspeed and midline, events are simply the significant peaks or wells, with amplitude above or below a certain threshold, the latter being specifically tuned for each type of function. Events in the crabspeed function are used to detect rolls and combinations of events in the length, width and head angle are used to detect hunches and head casts.

Detection of events in the speed signal, which highly oscillates due to peristaltic nature of larval crawling, is performed using a different approach, as described below. In either case, event detection is completed by computing an event signal, which is nonzero at events (equals the event amplitude) and zero outside the events, and by storing the event characteristics (see Fig. 2B for example of automatically detected events).

#### Event detection in a non-oscillating signal

Our procedure is an extension of the “Schmitt trigger” algorithm previously used for detection of movement events in flies [36]. The extended algorithm employs four, rather than two, adjustable thresholds, which are specifically tuned for each type of signal. The thresholds are: 1) the upper and 2) the lower amplitude threshold; 3) the width threshold; and 4) the gap threshold. An event starts when the absolute value of a signal, while increasing as a function of time, crosses the upper amplitude threshold. An event ends when the absolute value of a signal, while decreasing as a function of time, crosses the lower amplitude threshold. Upon detection of events, the algorithm stores its duration, amplitude and frequency. Event duration is the difference between the event end and event start times. Event amplitude is the highest absolute value of a function during the event. A single event of duration less than the width threshold will not be detected (i.e., will be ignored). However, if two or more adjacent events of the same type (all peaks or all wells) are less than the gap threshold apart one from another, and the time duration between the start of the first event and the end of the last event exceeds the width threshold, then all the events will be merged into a single detected event. Finally, event frequency is defined as a count of events (e.g. rolls, casts or hunches) detected within a given time interval.

The thresholds for each function were set as follows. Ground truth data for each action was manually labeled to indicate whether the larva was performing an action at the time or not by a human expert. 30 examples of larvae performing the action and not performing the action were used. For each labeled action the value of the peak or well of the relevant function was determined. Initial thresholds were then set based on this data and the number of false positives and false negatives detected by the algorithm in the ground truth data, relative to the human expert was compared. The thresholds were readjusted and the process was iterated several times, until false detection rate in the ground truth data was less than 5% and false negative rate was less than 15%. The algorithm was then tested on “novel” data, which was also ground-truthed by a human expert and the final false positive and false negative rates were calculated.

#### Detection of rolls

Rolling is the simplest example of action detection, as it only requires processing of a single variable, the crabspeedEach significant peak in the crabspeed function corresponds to a roll. The following thresholds are used: upper amplitude threshold, 2.8 mm/s; lower amplitude threshold, 1.8 mm/s; width threshold, 0.12 s and gap threshold 1 s. False detection rate = 3.6% (N = 28).

#### Detection of head casts and hunches

Accurate detection of the head casts and hunches requires simultaneous use of several variables: head angle, spine length, width, x, and y (see Supplementary software for details). However, a peak or a well in the head angle function generally corresponds to a head cast. In particular, a peak in the head angle function will typically correspond to a left head cast, while a well in the head cast function will typically correspond to a right head cast. The following head angle thresholds are used: upper amplitude threshold 27°, lower amplitude threshold 20°, width threshold 0.15 s and gap threshold 0.67 s. A well in the length function generally corresponds to a hunch. The following midline thresholds are used: upper amplitude threshold 0.19 mm, lower amplitude threshold 0.09 mm, width threshold 0.2 s and gap threshold 0.3 s. False detection rate for head cast and hunch was 3.9% (N = 77) and 2.5% (N = 35), respectively.

#### Event detection in an oscillating function: peristaltic crawling strides and runs

Run detection procedure is initiated by identifying peaks in the oscillatory speed function, that correspond to peak speed of individual crawling strides. Peak positions are the local maxima of the speed function. Peak amplitude is the function value at the peak position. Peak boundaries are set at the minima of the function on both sides of a peak. A peak is considered good if its amplitude exceeds the fixed threshold 0.6 mm/s and, at the same time, is at least 3/10 of the mean peak amplitude computed across an entire speed trace for a given animal. A crawling run is a sequence of at least three adjacent good peaks. A crawling run is terminated if the gap between two adjacent good peaks exceeds 2 s. Crawling runs are also terminated at rolls and head cast actions. False detection rate of crawling runs was 3.1% (N = 32). Upon detection of a run, the algorithm stores its duration, mean maximum stride speed and stride frequency. Run duration is the difference between the event boundaries. Mean maximum stride speed of a run is the mean height of all the good peaks comprising the run. Event boundaries are set at the boundaries of the first and last peak comprising the event. The stride frequency of a run is determined by applying spectral analysis to the portion of signal representing the event. For our unevenly sampled data, this procedure is performed using an implementation of the Lomb-Scargle algorithm [37] (pages 685–699) in R programming language.

#### Performing statistical tests

Binary statistical tests implemented in R programming language [38] were used to compare the features extracted from detected behavioral actions across different animal lines. Each test analyzed one feature at a time. For each feature only those animals were considered that spanned the entire time interval of interest. Animals that only partially spanned the interval, because they crawled to the edges or bumped into each other and were lost to tracking in the course of the time interval were not considered.

For the features with continuous distribution, such as head cast amplitude, mean crawling speed, stride speed and stride frequency, the non-parametric Wilcoxon’s rank sum test was used. The probability of an action (roll, cast, hunch, crawl probability) for a given genotype was computed as a percentage of animals that performed the action within the time window (out of all the animals whose tracking time spanned the entire interval). Binary statistical tests available for proportions were used to compare genotypes. More specifically, for each considered time interval, a 2×2 contingency table was first built by counting, for either animal line, the total number of tracked animals and the number of animals that participated in at least one action of the specified type. Depending on whether or not all the elements of the contingency table were >5, we used Fisher’s exact test and Chi Squared Test, respectively (see Tables S1, S2, S3, S4 for all values).

## Results

### Hardware and Software for Analysis of Larval Somatosensation

We developed hardware modules for delivering distinct somatosensory and optogenetic stimuli to many larvae at once while tracking their behavior with a video camera (Fig. 1A) (detailed plans will be available on HHMI Janelia website upon publication).

To study larval reactions to noxious thermal stimuli, we developed a module that uses infrared (IR) solid-state lasers to deliver light (808 nm) across arena. The laser light itself does not induce escape behaviors because larvae do not strongly absorb this wavelength. To induce localized noxious heat, a black dot is painted on each larva with a permanent marker (Sharpie, Rub a Dub). We were able to induce rolling in around 30 larvae at once by using a 1 s pulse of IR light (Video S1). The dot can be painted on the dorsal wall or on the side of the body (Fig. 1B and 1C). In the latter case, all larvae roll toward the painted side in response to light (Video S2).

For investigating responses to mechanosensory stimuli and to optogenetic activation of neurons, we designed three stimulus delivery modules for a large 25×25 cm^2^ arena. Air-current is delivered through a flare nozzle connected to plant-supplied compressed air at a pressure of up to 1.4 MPa. We could generate uniform rates across the arena (±0.9 m/s) within the range of air-flow rates from 2 to 7 m/s. We also improved our previously described module for presenting vibration so that it could generate vibrations across the large arena anywhere between 0.1 and 2 kHz. The frequencies specified by the signal generator matched the measured dominant agar vibration frequencies (Fig. 1D and 1E). To stimulate or inhibit larval neurons with optogenetic tools, we designed an array of LEDs to cover the arena uniformly with 470 nm light of intensity of 0.17 mW/mm^2^.

All of the stimulus modules are controlled through the previously described Multi-Worm Tracker software (MWT) [35]. Video microscopy of larvae within the experimental arena was done with a DALSA Falcon 4M30 camera and contours of larvae as a function of time were extracted from the video with the MWT software at temporal resolution of up to 25 fps. To quantify larval responses to somatosensory stimuli from the movies of contours, we developed custom signal-processing software, LARA, for automated detection of a comprehensive repertoire of larval motor patterns. To do so we first visually examined the movies of contours of hundreds of larvae and noted the motor patterns that they performed in response to somatosensory stimuli. Due to the high temporal resolution of the data we could observe periodic peristaltic crawling strides [7] as oscillations in the speed of motion of the center of mass. During a stride a peristaltic wave of muscle contractions propagates from posterior to anterior segments and then the anterior is propelled forward resulting in a sudden increase in the speed of center of mass [39]. We observed that larvae could modulate the maximum speed with which the anterior is propelled forward (stride speed) and the stride frequency. We also observed alterations in the duration of uninterrupted linear crawling bouts (runs). Other motor patterns we observed included head retractions (hunches), head casts and rolls. We defined one or more functions that could be used by LARA to detect each motor pattern in an automated way (Fig. 2B). For example, oscillations in the forward speed of the center of mass are used to detect strides, large increases in the sideways speed of the center of mass (crabspeed) are used to detect rolls and a combination of alterations in the length and width of the animal and head angle are used to detect head retractions and head casts. In this way, LARA detects and quantifies the different motor patterns recorded in the videos with a false detection rate of less than 5% (see Materials and Methods for further details). LARA can output amplitude, probability and duration of each motor pattern (Fig. 2E) and generate behavior raster plots of larval behavior (Fig. 2C) or population average plots (Fig. 2D).

### Thermal Noxious Stimulation Evokes a Dynamic Sequence of Escape Reactions

**Figure 3:**
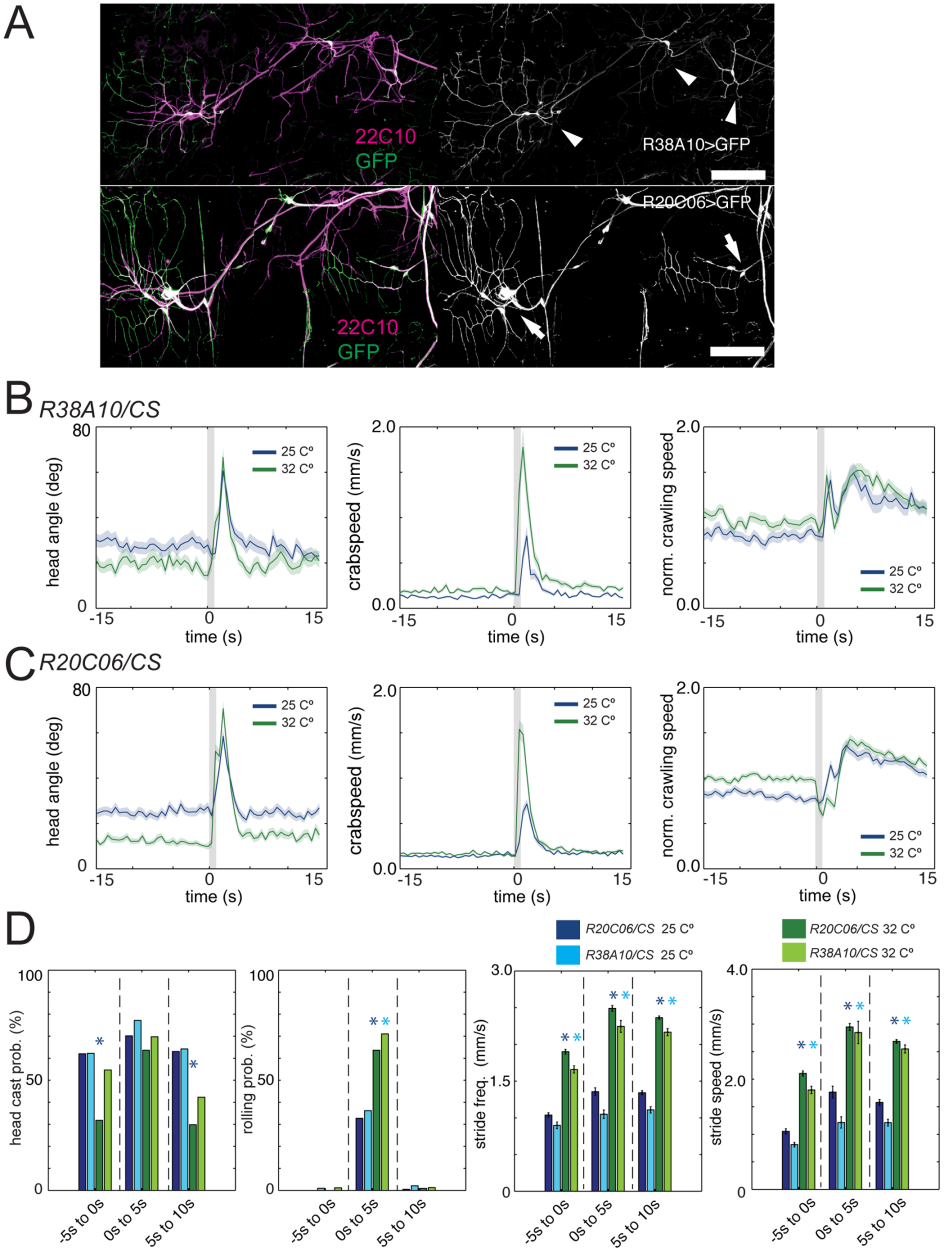
Noxious heat reactions are modulated by ambient temperature. (A) Expression patterns of *R38A10* and *R20C06*. Confocal microscope images of the A3 hemisegment of third instar *R38A10>GFP* and *R20C06>GFP* larvae. Larvae are co-immunostained with antibodies against GFP (green, left; white, right) and 22C10, a marker of all peripheral sensory neurons (magenta, left). Anterior is up. Dorsal midline is to the right. Scale bar represents 100 µm. *R38A10* drives expression in class IV neurons (arrowheads) and *R20C06* in class I md neurons (arrows) (compare to the reference images in Grueber et al. 2002 [54]). (B and C) Graphs show head angle, crabspeed and normalized crawling speed as a function of time as in Fig. 2D. Dark lines, mean value. Light lines, ± s.e.m. Grey lines at 0 s mark the stimulus onset and duration. Data from control *R38A10*>*Canton S* and *R20C06>Canton S* at 32°C (green, N = 91 and 102, respectively) and 25°C (blue, N = 98 and 184, respectively) is compared. The increase in the mean crabspeed function following stimulation (corresponding to the roll) is present under both conditions, but it is both faster and larger at 32°C compared to 25°C. Escape crawl looks similar under both conditions (the increase in mean speed in response to noxious stimulation, relative to mean speed prior to stimulation), although the absolute crawling speed is drastically different (see below). (D) Bar charts show head casting and rolling probability and the mean value of the maximum stride frequency and stride speed as in Fig. 2E. Error bars indicate s.e.m. * (light blue star) and * (dark blue star), *p*<0.001 for *R38A10*>*Canton S* and *R20C06>Canton S,* respectively, when behavior at 32°C is compared to 25°C in the same time window. Rolling probability of *R38A10*>*Canton S* (light green) and *R20C06>Canton S* (dark green) is drastically increased at 32°C compared to 25°C (light blue and dark blue) (from 36% and 32% to 71% and 63.6%, N = 105, 171, 76, 55; *p = *0.000063 and 0.000846). Likewise, stride frequency and stride speed are significantly increased at 32°C compared to 25°C, both prior to stimulation and following stimulation (see Table S1 for further details).

**Figure 4:**
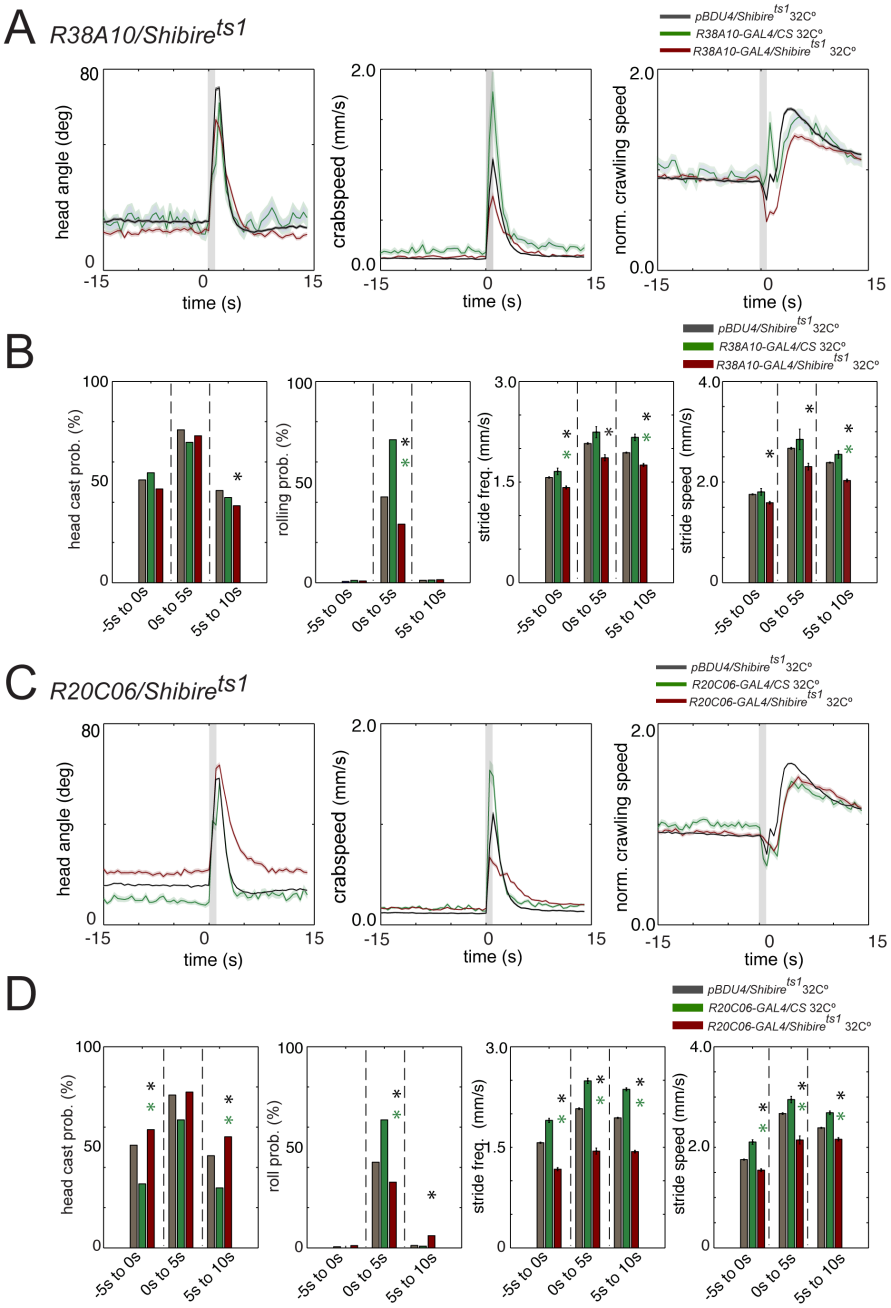
Noxious heat reactions are altered when class IV or class I neurons are inactivated. (A and C) Graphs show head angle, crabspeed and normalized crawling speed as a function of time as in Fig. 2D. Dark lines, mean value. Light lines, ± s.e.m. Grey lines at 0 s mark the stimulus onset and duration. Data from control *pBDPUGAL4>shibire^ts1^* (black, N = 8461) and *R38A10*>*Canton S* or *R20C06>Canton S* at 32°C (green, N = 76 and 55, respectively) and *R38A10>shibire^ts1^* or *R20C06>shibire^ts1^* (red, N = 915 and 1144, respectively) is compared. (B and D) Bar charts show head casting and rolling probability and the mean value of the maximum stride frequency and stride speed as in Fig. 2E. Error bars indicate s.e.m. * (black star), *p*<0.001 when compared to *pBDPUGAL4>shibire^ts1^* in the same time window. * (green star), *p*<0.001 when compared to *R38A10*>*Canton S* or *R20C06>Canton S* in the same time window. Rolling probability of *R38A10>shibire^ts1^* (B, red, 29.1%, N = 915) is drastically reduced compared to *pBDPUGAL4>shibire^ts1^* (B, black, 42.6%, N = 8461; *p*<10^−6^) and *R38A10*>*Canton S* (B, green, 71.1%, N = 76; *p*<10^−6^) in the time window following noxious heat stimulation. Likewise, stride frequency and stride speed are significantly decreased compared to controls, both prior to stimulation and following stimulation (see Table S1 for further details). Rolling probability of *R20C06>shibire^ts1^* (D, red, 32.7%, N = 1144) is drastically reduced compared to *pBDPUGAL4>shibire^ts1^* (D, black, 42.6%, N = 8461; *p*<10^−6^) and *R20C06*>*Canton S* (D, green, 63.6%, N = 55; p = 0.000045) in the time window following noxious heat stimulation. Likewise, stride frequency and stride speed are significantly decreased compared to both controls, both prior to stimulation and following stimulation (see Table S1 for further details), consistent with the role of these neurons in proprioception.

We described in detail larval reactions to 1 s long noxious heat stimuli by analyzing behavior raster plots and population average curves obtained from wild-type *Canton S* larvae that did not contain any transgenes and from genetic background control larvae for future neural inactivation experiments (*R38A10/Canton S*, R20C06*/Canton S*, pBDPGAL4U*/UAS-shibire^ts1^*) [2], [29] (Fig. 2, 3 and 4). We intend this work to be the basis for performing neural inactivation screens with the recently generated sparse collection of *GAL4* driver lines [1] that are all inserted in the same site in the genome. The *pBDPGAL4U* line has the same vector inserted in the same site in the genome as the other *GAL4* driver lines, but it lacks a cell-type specific enhancer, resulting in no expression of the GAL4 [23]. Crossed to the effector of choice it serves as an ideal control for the *GAL4* collection. Since we planned to perform inactivation experiments at restrictive temperature for Shibire^ts1^ in the future we performed all the initial wild-type behavior characterization experiments at 32°C.

Prior to noxious heat stimulation, larval behavior generally consisted of peristaltic crawling interrupted by exploratory head casts (Fig. 2A, 2B and 2C, Videos S1, S2, S3, and S4). Noxious heat evoked twisting (head bending) and rolling, as previously described [9] (Fig. 2, Videos S1, S2 and S3). We found that larvae could roll with speeds up to 8 mm/s (mean maximum rolling speed of 4.66 mm/s, N = 6,635, for *pBDPGAL4U/UAS-shibire^ts1^* and 4.56 mm/s, N = 96, for Canton S), consistent with previous reports [9], [13].

Our high-resolution analysis also revealed a novel component of the escape response to noxious heat that had not been described before, which we called *escape crawl*. This behavior was clearly detectable both in *pBDPGAL4U/UAS-shibire^ts1^* and in *Canton S* larvae. We found that after rolling, animals resumed crawling at speeds that were on average 1.5 times higher than before simulation and higher than in response to any other stimulus (Fig. 2D and 2E). Crawling was fastest 4 s after stimulation and slowly returned to baseline by 15 s after stimulation (Fig. 2D). Larvae achieved this increase in speed by increasing stride speed and stride frequency (Fig. 2E and Table S1). We found that rolling itself was not necessary for *escape crawling* and that some larvae initiated *escape crawling* in response to noxious heat without rolling first (25%). Taken together our analysis shows that noxious heat evokes several kinds of escape behaviors comprised of specific sequences of motor patterns, the most vigorous one being *bend-roll-escape crawl* and an alternative being *bend-escape crawl*.

### Identifying Defects in Larval Reactions to Noxious Thermal Stimuli

Next we asked whether our method could be used to identify neurons required for larval escape responses to noxious thermal stimuli. By using the GAL4/UAS system [28], we can acutely block neurotransmission in classes of somatosensory neurons previously implicated in rolling behavior with *UAS-Shibire^ts1^* at restrictive temperature (32°C). We selected two GAL4 lines, *R38A10* and *R20C06* that drove expression in the nociceptive class IV neurons, and the proprioceptive class I neurons, respectively (Figure 3A). The ideal control for such inactivation experiments is *pBDPGAL4U/UAS-shibire^ts1^* at restrictive temperature as both the genetic background and the temperature conditions are constant across all experiments. *R38A10/Canton S* and *R20C06/Canton S* at restrictive temperature can serve as individual GAL4 controls, since *Canton S* is the genetic background of the *UAS-Shibire^ts1^* lines we are using. A further possible control is to compare the behavior of *R38A10/UAS-Shibire^ts1^* and *R20C06/Shibire^ts1^* larvae at restrictive temperature (32°C) with the behavior of these larvae at the permissive temperature (25°C), keeping the genetic background constant, but the temperatures conditions different. The caveat with this last control is that the behavior of larvae at the two temperatures could be so different that a comparison would not make sense. To assess the feasibility of using the permissive temperature control we compared the reaction of the control larvae, *R38A10/Canton S* and *R20C06/Canton S* larvae to noxious heat at 32°C and 25°C. We found that while the essential elements of the reaction (roll and escape crawl) were present at both temperatures there was a drastic increase in rolling probability and in the absolute crawling stride speed and frequency at 32°C, relative to 25°C (Fig. 3B–3D). These findings reveal an interesting context-dependence of the larval reaction to noxious heat. The drastic context-dependence makes the use of the permissive-temperature control impossible in our inactivation experiments and points to the importance of comparing all the genotypes at constant environmental conditions during the experiment.

To identify the effect of inactivation of class IV and class I neurons on larval escape responses to noxious heat, we therefore compared *R38A10/UAS-Shibire^ts1^* and *R20C06/Shibire^ts1^* larvae at restrictive temperature (32°C) with the behavior of *pBDPGAL4U/UAS-shibire^ts1^* and *R38A10/Canton S* or *R20C06/Canton S* larvae at the same temperature (Fig. 4).

We observed a highly significant reduction in rolling in response to noxious stimuli compared to both controls when we inactivated the nociceptive class IV neurons (Chi square test, *p<*10^−6^, Fig. 4A and 4B and Table S1) [2], [29], [40]. Larvae with inactivated class IV neurons were also impaired at *escape crawling* following noxious stimulation (Fig. 4A and 4B). Stride speed and frequency were significantly lower compared to controls (Fig. 4B). Surprisingly, stride speed and frequency were reduced compared to controls even prior to stimulation (Fig. 4B and Table S1). When we inactivated the proprioceptive class I md neurons (*R20C06/Shibire^ts1^*) we observed a dramatic reduction in the absolute stride speed and frequency (Fig. 4C and 4D and Table S1), both before and after stimulation, compared to controls. We also observed a significant reduction in rolling events immediately following noxious stimulation (Fig. 4D). These results are consistent with previous studies that implicated the class I neurons in proprioceptive feedback [13], [41].

**Figure 5:**
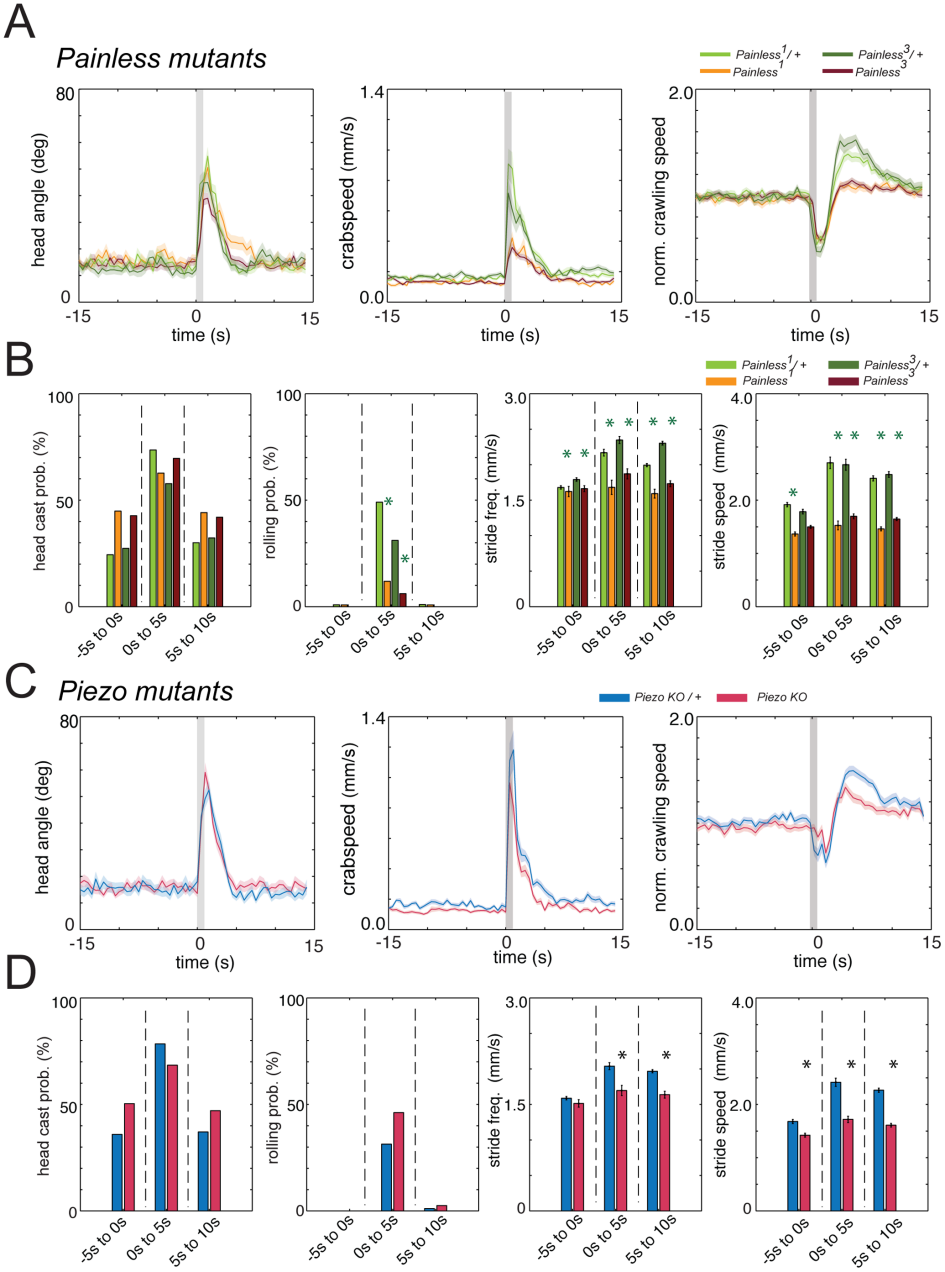
*painless* mutant larvae are impaired in rolling responses to noxious heat. (A and C) Graphs show head angle, crabspeed and normalized crawling speed as a function of time, as in Fig. 2. Dark lines, mean value. Light lines, ± s.e.m. Grey lines at 0 s mark stimulus onset and duration. Data from the control *painless^1^>w1118* (light green, N = 53), *painless^3^>w1118* (dark green, N = 45) and *piezoKO>w1118* (blue, N = 51) is compared to *painless^1^* (orange, N = 126), *painless^3^* (red, N = 181) and *piezoKO* (red, N = 130) mutants, respectively. In both *painless^1^* and *painless^3^* mutants the peaks in the mean crabpseed and the mean normalized speed functions are highly reduced compared to controls. They show virtually no *escape crawl*. (B and D) Bar charts show head casting and rolling probability and the mean value of the maximum stride frequency as in Fig. 2E. Error bars indicate s.e.m. * (light green star), * (dark green star) and * (blue star) indicate *p*<0.001 when *painless^1^*, *painless^3^* and *piezoKO* is compared to *painless^1^>w1118, painless^3^>w1118* and *piezoKO>w1118*, respectively. In response to noxious heat stimulus, the rolling probability of *painless^1^* (11.9%, N = 126) and *painless^3^* (6.1%, N = 181) larvae, defective in thermal nociception, is significantly reduced compared to the hemizygous controls (49.1%, N = 53, p<10^−6^ and 31.1%, N = 45, p = 0.000054). The mutants also have significantly reduced stride frequency and stride speed following stimulation and reduced stride frequency prior to stimulation (see Table S1 for further details). In contrast, *piezoKO* mutant larvae defective in mechanical nociception roll slightly, but not significantly more than the hemizygous controls. Interestingly they are significantly defective in escape crawl and in stride speed prior to stimulation.

We also confirmed that our method could be used to identify genes important for these behaviors. We tested larvae mutant for two ion channels, *painless* and *Dmpiezo*, previously shown to be required for thermal [9] and mechanical [19] nociception, respectively. As expected, we detected significantly less rolling and a drastic reduction in escape crawling in *painless^1^* and *painless^3^* mutant larvae compared to the hemizygous controls (Fig. 5A and 5B). Both mutants also showed mild defects in baseline crawling, relative to the controls. In contrast, *Dmpiezo* mutant larvae rolled more than the controls, even though this increase was not significant (Fig. 5D). Surprisingly *Dmpiezo* mutant larvae were impaired in baseline crawling and in escape crawling (Fig. 5C and 5D). This confirms previous findings that *Dmpiezo* is not required for rolling in response to the thermal nociceptive stimulus, but only in response to a harsh mechanical stimulus. However all three mutants also showed defects in baseline crawling, possibly due to additional genetic background effects or due to developmental effects of these mutations. These results also demonstrate that crawling and rolling can be affected independently from each other.

### Vibration is Sensed by ch Neurons and Evokes a Dynamic Sequence of Reactions

**Figure 6:**
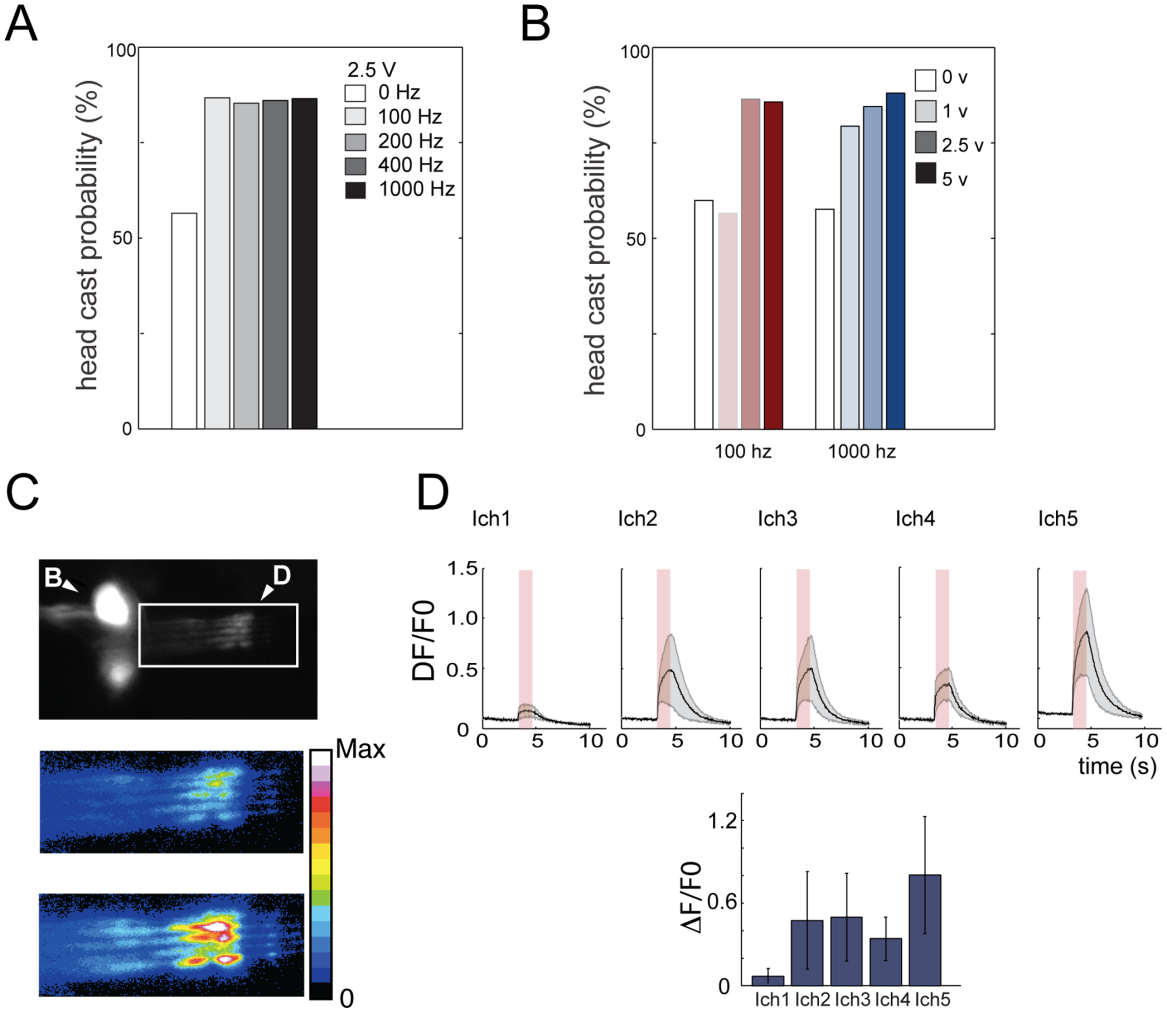
Vibration evokes head-casting and is sensed by chordotonal neurons. (A and B) Larval reaction to a range of vibration frequencies and intensities. Bar charts show head cast probability in a 5 s time window following vibration onset compared to a 5 s time window in the absence of vibration (0 Hz and 0 V, white bar). (A) Larvae significantly increase head cast probability compared to the baseline prior stimulation, in response to a range of frequencies from 100 Hz to 1000 Hz. N equals 92, 76, 104, 100 and 111 for 0 Hz, 100 Hz, 200 Hz, 400 Hz and 1000 Hz. *p*<10^−6^, for 0 Hz, compared to 100 Hz, 200 Hz, 400 Hz and 1000 Hz, respectively. (B) Head cast probability increases as voltage applied to the speaker increases, at 100 Hz and at 1,000 Hz, reaching the peak reaction of about 90%. N equals 67, 63, 76, 84, 105, 112, 111 and 105, respectively. *p*<10^−6^, for 100 Hz, 2.5 V and 5 V compared to 0 V and for 1000 Hz, 1 V, 2.5 V and 5 V, compared to 0 V. (C and D) Larval lateral ch (lch1-5) neurons sense 1000 Hz vibration. (C) An image of Ca^2+^ signals visualized with GCaMP3 in the dendrites (inside white rectangle) of lch1-5 in one abdominal hemisegment (A4), before stimulation (top and middle) and during a 1000 Hz, 2 V tone (1 sec after stimulus onset) (bottom). B, cell body cluster of lch1-5. D, dendrites of lch1-5. Anterior is up. Dorsal midline is to the right. Color code (middle and bottom panels), pseudocolored fluorescence intensity levels using the Fiji 16 color code [34]. White and black, highest and lowest intensity, respectively. (D) Quantification of GCaMP responses in the five individual members of the lch1-5 cluster in A4. Graphs show mean ΔF/F_0_ in the dendrite of each ch neuron. Error bar represent s.e.m. N = 4 larvae. Different members of the lch1-5 cluster have differential sensitivity to 1000 Hz, the most sensitive being lch5, lch2, lch3 and lch4, and least sensitive lch1.

Next we applied our system to a detailed characterization of larval responses to mechanical stimuli. Recently we have shown that *Drosophila* larvae react to 1,000 Hz vibration by head casting [11] and hunching [12], but a detailed analysis of larval sensitivity and reactions to vibration was lacking. With our new speaker module we now tested head casting responses to a range of frequencies and amplitudes (Fig. 6A and 6B). We observed reactions to frequencies from 100 Hz to 1,000 Hz (Fig. 6A) and found that the probability (Fig. 6B) and amplitude (data not shown) of head casting varied in an intensity-dependent manner.

Previously we have also shown that larval ch neurons were required for normal head casting and hunching to vibration [11], [12], but we had not performed any physiological studies to test whether ch neurons are indeed mechanosensory neurons, activated by vibration. We therefore performed calcium imaging experiments in ch neurons in response to 1,000 Hz tones (Fig. 6C and 6D, Video S5). We observed large calcium transients in response to 1,000 Hz tones in four members of the lateral ch cluster (lch1-5) (Fig. 6D).

**Figure 7:**
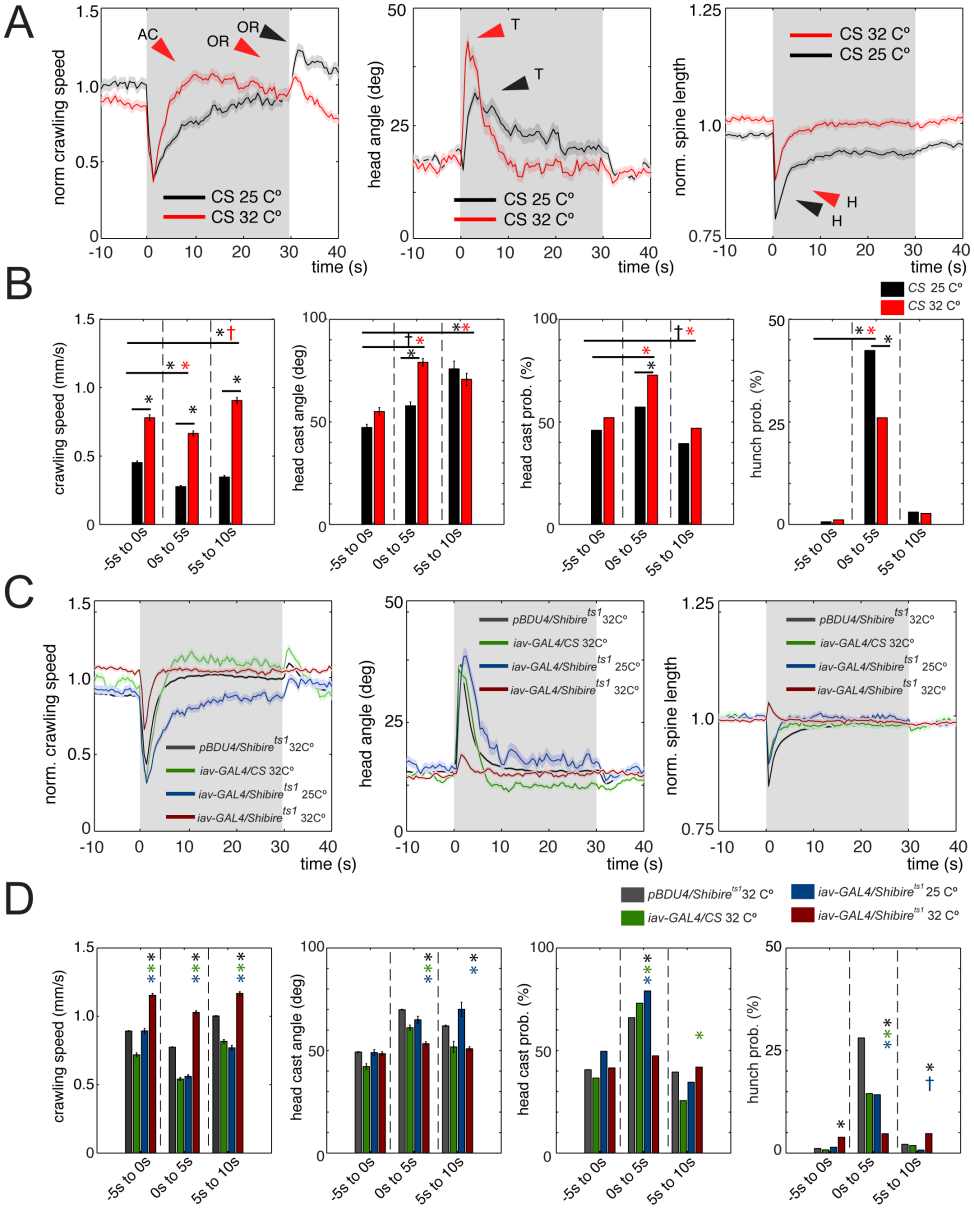
Detailed characterization of the larval reactions to vibration and the role of chordotonal neurons. (A and B) Vibration evokes a characteristic dynamic sequence of behaviors. (A) Graphs of mean normalized crawling speed, head angle, and normalized spine length as a function of time averaged across many animals from experiments in which wild-type *Canton S* (CS) larvae were presented with 30 s of continuous vibration (1000 Hz, 2 V) at ambient temperature of 32°C (red, N = 342) or 25°C (black, N = 248). Normalized crawling speed was computed as in Fig. 2D. Gray shading indicates the period of stimulation. Dark lines, mean value. Light lines, ± s.e.m. AC, *avoidance crawl*. OR, *avoidance crawl off-reaction* by speeding up. T, head cast (turn). H, hunch. Graphs highlight the dynamics of the reaction to vibration. Following vibration onset, there is a sharp well in the norm. spine length function, corresponding to the hunch (H), then a sharp peak in the head angle function (T), corresponding to the increase in head casting and turning. As these two functions return to baseline there is a raise in the speed function as larvae start crawling again. At 32°C the mean speed during vibration raises significantly above the speed prior to stimulation – indicating larvae are trying to actively avoid vibration by crawling faster (*avoidance crawl*). Following vibration offset there is significant increase in crawling speed relative to the baseline prior to stimulation at both 32°C and 25°C (*avoidance crawl off-reaction*, OR). Interestingly, while *avoidance crawling* in response to vibration offset happens at both temperatures, *avoidance crawl* during vibration only happens at 32°C, but not at 25°C. The precise nature of the reaction to vibration, like the reaction to noxious stimulation, is highly context-dependent. (B) Bar charts show the mean absolute larval crawling speed, the mean maximum head angle during head casts and the head casting and hunching probability in a 5 s time window before stimulation (−5 s to 0 s) and in two consecutive 5 s time windows after stimulation (0 s to 5 s and 5 s to 10 s). Error bars indicate s.e.m. * and *, *p*<0.001.^+^and ^+^, *p*<0.01. The mean absolute larval crawling speed is significantly higher at 32°C than at 25°C, in all three time windows. At 32°C, but not at 25°C, the absolute mean crawling speed is higher in the 5 s to 10 s, than in the 0 s to 5 s window indicating that *avoidance crawl* during stimulation only happens at the higher temperature (see Table S2 for further details). Head cast angle and probability are higher at 32°C than at 25°C, whereas hunch probability is higher at 25°C than at 32°C. Even though the reactions to vibration are significantly different at different temperatures, many aspects of the reaction are pronounced enough at 25°C to allow the use of the permissive temperature *UAS-Shibire^ts1^* control. (C and D) Ch neurons are implicated in most aspects of the larval reaction to vibration. (C) Graphs of mean normalized crawling speed, head angle, and normalized spine length as in A at 32°C. Gray shading indicates the period of stimulation. Dark lines, mean value. Light lines, ± s.e.m. Data from larvae with inactivated ch neurons (red, *iav>shibire^ts1^* at restrictive temperature of 32°C, N = 820) is compared to three different kinds of control larvae. Blue, *iav>shibire^ts1^* at permissive temperature of 25°C (N = 299). Green, *iav>Canton S* at 32°C (N = 457). Black, *pBDPGAL4U>shibire^ts1^* at 32°C (N = 24,865). Most aspects of the reaction to vibration are compromised in larvae with inactivated ch neurons, compared to controls. Avoidance crawl and off-reaction in the normalized speed function are not visible. The peak in the head angle function is drastically reduced. The well in the norm. spine length function is gone and instead a small peak is visible – indicating that the residual reaction to vibration that is left is actually opposite in sign and abnormal. (D) Bar charts show the mean absolute larval crawling speed, the mean maximum head angle during head casts and the head casting and hunching probability as in B. Error bars indicate s.e.m. * (blue star), * (green star) and * (black star) indicate *p*<0.001 when *iav>shibire^ts1^* at 32°C is compared to *iav>shibire^ts1^* at 25°C, *iav>Canton S* at 32°C and *pBDPGAL4U>shibire^ts1^* at 32°C, respectively. The magnitude of the head cast angle and the head cast and hunch probability following stimulation are significantly reduced in *iav>shibire^ts1^*, compared to all three controls (see Table S2 for further details).

Next we wanted to characterize in more detail the dynamics of the response of wild-type *Canton S* larvae to 30 s of continuous 1,000 Hz, 2 V vibration, at two different ambient temperatures (25°C and 32°C). We identified a characteristic dynamic sequence of reactions with new behaviors, in addition to the previously described head cast [11] and hunch [12] (Fig. 7A and 7B, Table S2, Video S6). Furthermore, we observed an interesting modulation of the reaction to vibration by temperature. At 32°C, immediately following vibration onset, larvae pause and hunch and then head cast (Fig. 7A and 7B). After the brief head-casting phase, larval crawling speed raises slightly, albeit significantly above the baseline before stimulation (Fig. 7A and 7B). This phase may represent active non-directional avoidance (*avoidance crawl*) of the stimulus. We also observed a further increase in crawling speed (*avoidance crawl*) in response to stimulus offset (*off reaction*) (Fig. 7A). Interestingly, at 25°C only the *avoidance crawl* in response to vibration offset was present, but not during continuous vibration (Fig. 7A and 7B). At 32°C the mean crawling speed was higher during, than prior to vibration, but at 25°C it was lower during, than prior to vibration. Mild heat (32°C) also increased crawling speed in the absence of vibration, but a combination of vibration and mild heat resulted in even higher crawling speeds (Fig. 7A and 7B). This can be summarized with the equation below:
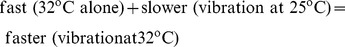



Thus, combining mild heat with vibration results in a “sign-reversal” of the speed response.

Other motor patterns evoked by vibration were also affected by temperature. The head angle and head cast probability were higher at 32°C compared to 25°C, whereas hunching probability was higher at 25°C compared to 32°C (Fig. 7B). This complex temperature-dependence of the response provides and excellent basis for studying the basis of multi-modal integration.

### Ch Neurons are Implicated in most Aspects of the Reaction to Vibration

We have previously shown that ch neurons are required for head casting and hunching to vibration [11], [12], but their role in off-reactions and avoidance crawl has not been investigated. We used *iav-GAL4* to inhibit activity in all larval ch neurons [31], acutely with *UAS-Shibire^ts1^* at restrictive temperature (32°C) and analyzed multiple aspects of larval reactions. We used three kinds of controls. Even though the *wild-type* reactions to vibration at 25°C and 32°C were different, some aspects were similar enough to use the *iav-GAL4*/*UAS-Shibire^ts1^* control at permissive temperature (25°C). We also used the no-GAL4 control (*pBDPGAL4U/UAS-shibire^ts1^*) and the *iav-GAL4*/*Canton S* control. We found that larvae with inactivated ch neurons were significantly impaired in hunching and head casting in response to vibration, as previously described (Fig. 7C and 7D, Table S2). Inactivation of ch neurons also abolished the *avoidance crawl* in response to vibration offset (*off reaction*) and during vibration (Fig. 7C).

### Air Current Evokes a Distinct Characteristic Dynamic Sequence of Reactions

**Figure 8:**
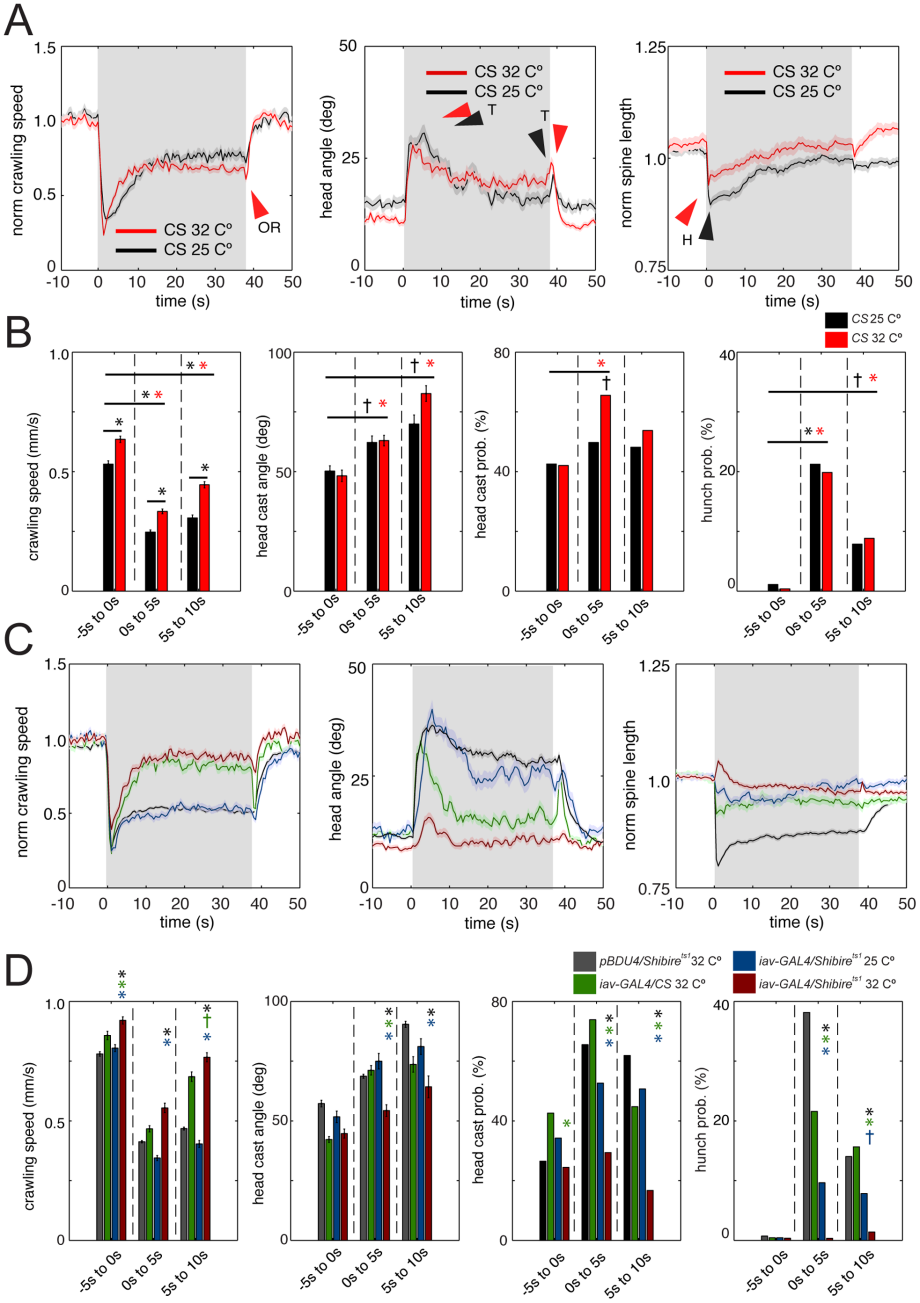
Detailed characterization of the larval reactions to air current and the role of chordotonal neurons. (A and B) Air current evokes a characteristic dynamic sequence of behaviors, distinct to vibration. (A) Graphs of mean normalized crawling speed, head angle, and normalized spine length as a function of time averaged across many animals from experiments in which wild-type *Canton S* (CS) larvae were presented with 45 s of continuous air current (6.5 m/s) at 32°C (red, N = 318) or 25°C (black, N = 201). Normalized crawling speed was computed as in Fig. 2D. Gray shading indicates the period of stimulation. Dark lines, mean value. Light lines, ± s.e.m. OR, off-reaction by slowing down. T, head cast (turn) at stimulus onset and offset. H, hunch. Following air current onset, there is a well in the norm. spine length function, corresponding to the hunch (H), followed by a peak in the head angle function (T), corresponding to the increase in head casting and turning. The reaction to air current at different temperatures is quite similar. Thus the reactions to vibration and air current are drastically different at 32°C. During vibration at this temperature larvae exhibit avoidance crawling, whereas during air current they slow down and continue hunching and turning. Furthermore the off-reactions to vibration and air current are opposite in sign, at both temperatures. In response to air current offset larvae slow down and head cast more, whereas in response to vibration offset they speed up (*avoidance crawl*) and head cast less. (B) Bar charts show the absolute larval crawling speed, the maximum head angle during head casts and head casting and hunching probability as in Fig. 7B. Error bars indicate s.e.m. * and *, *p*<0.001.^+^and ^+^, *p*<0.01. The mean absolute larval crawling speed is significantly higher at 32°C than at 25°C in all three time windows, but it is always lower during air current than prior to air current. Head cast probability during air current stimulation is higher at 32°C than at 25°C (see Table S3 for details). (C and D) Ch neurons are implicated in larval reaction to air current. (C) Graphs of mean normalized crawling speed, head angle, and normalized spine length as in A at ambient temperature of 32°C. Gray shading indicates the period of stimulation. Dark lines, mean value. Light lines, ± s.e.m. Data from larvae with inactivated ch neurons (red, *iav>shibire^ts1^* at restrictive temperature of 32°C, N = 362) is compared to three different kinds of control larvae. Blue, *iav>shibire^ts1^* at permissive temperature of 25°C (N = 242). Green, *iav>Canton S* at 32°C (N = 296). Black, *pBDPGAL4U>shibire^ts1^* at 32°C (N = 1616). Some aspects of the reaction to air current are more compromised than other in larvae with inactivated ch neurons. The peak in the head angle function is drastically reduced. The well in the norm. spine length function is gone and instead a small peak is visible. (D) Bar charts show the mean value of the absolute larval crawling speed, the mean maximum head angle during head casts and the head casting and hunching probability as in B. Error bars indicate s.e.m. * (blue star), * (green star) and * (black star) indicate *p*<0.001 when *iav>shibire^ts1^* at 32°C is compared to *iav>shibire^ts1^* at 25°C, *iav>Canton S* at 32°C and *pBDPGAL4U>shibire^ts1^* at 32°C, respectively. The magnitude of the head cast angle and the head cast and hunch probability following stimulation are significantly reduced in *iav>shibire^ts1^* at 32°C, compared to all three controls (see Table S3 for details).

We asked whether larvae reacted to different mechanical stimuli in different ways. *Drosophila* adults react to air currents as well as to sound [42], but whether larvae could react to air currents has previously not been investigated. We observed larval reactions to a range of air currents from light air (2 m/s) to fresh breeze (10 m/s). Air current of intensities higher than 10 m/s resulted in some larvae being blown off the assay plate, so we chose to work with a moderate breeze of 6.5 m/s. We analyzed in detail the reactions of wild-type *Canton S* larvae to a 37 s long air current stimulus at 25°C and 32°C. We found that air current modulated the same motor patterns as 1000 Hz vibration, but in different ways (Video S7, Fig. 8A and 8B, Table S3). While 1000 Hz vibration at 32°C, evoked avoidance crawling, air current decreased the mean crawling speed, compared to baseline (Fig. 8A and 8B). The dynamics of the head casting and hunching responses were also different. At 32°C, the magnitude of the head casting response to vibration onset was initially large, but the duration was relatively short (10 s), whereas the head cast response to air current lasted throughout the duration of the stimulus (37 s) (Fig. 8A and 8B). Another interesting difference between the reaction to vibration and air current was in the sign of the *off reaction*. In response to vibration offset there was an increase in mean crawling speed and a decrease in mean head angle (Fig. 7A). In contrast, in response to air current there was a decrease in mean crawling speed and an increase in mean head angle (Fig. 8A).

The differences in the reactions to vibration and air-current could be due to activation of distinct mechanosensory receptors or due to differential patterns of activity in the same receptors.

### Ch Neurons are Involved in Reactions to Air Current

In the adult *Drosophila*, distinct populations of ch neurons, with different intrinsic response properties and projection patterns, sense sound and wind [43]. We therefore asked whether ch neurons are also involved in sensing wind in the larva. We used *iav-GAL4* to inhibit activity in all larval ch neurons [31], acutely with *UAS-Shibire^ts1^* and compared larval behavior to the three types of controls as for vibration. We found that larvae with inactivated ch neurons were significantly impaired in hunching and head casting in response to air currents compared to all three controls (Fig. 8C and 8D, Table S3). However these animals still paused both in response to air current onset and offset (Fig. 8C). Thus, at least for some aspects of the larval reaction to air current, additional sensory neurons may be involved in sensing this stimulus.

### Optogenetic Activation of ch Neurons Evokes Head Casting

We complemented our sensory neuron inactivation studies with optogenetic activation using Channelrhodopsin (ChR2) (Fig. 1). For these experiments we used two kinds of controls: no-GAL4 controls (*pBDPGAL4U/UAS-ChR2*), that were fed with food that contains retinal (the necessary co-factor for ChR2) and the no-retinal controls that had identical genetic background to the “experimental” larvae (*iav-GAL4/UAS-ChR2 and R38A10/UAS-ChR2*), only they were fed with the food without retinal.

**Figure 9:**
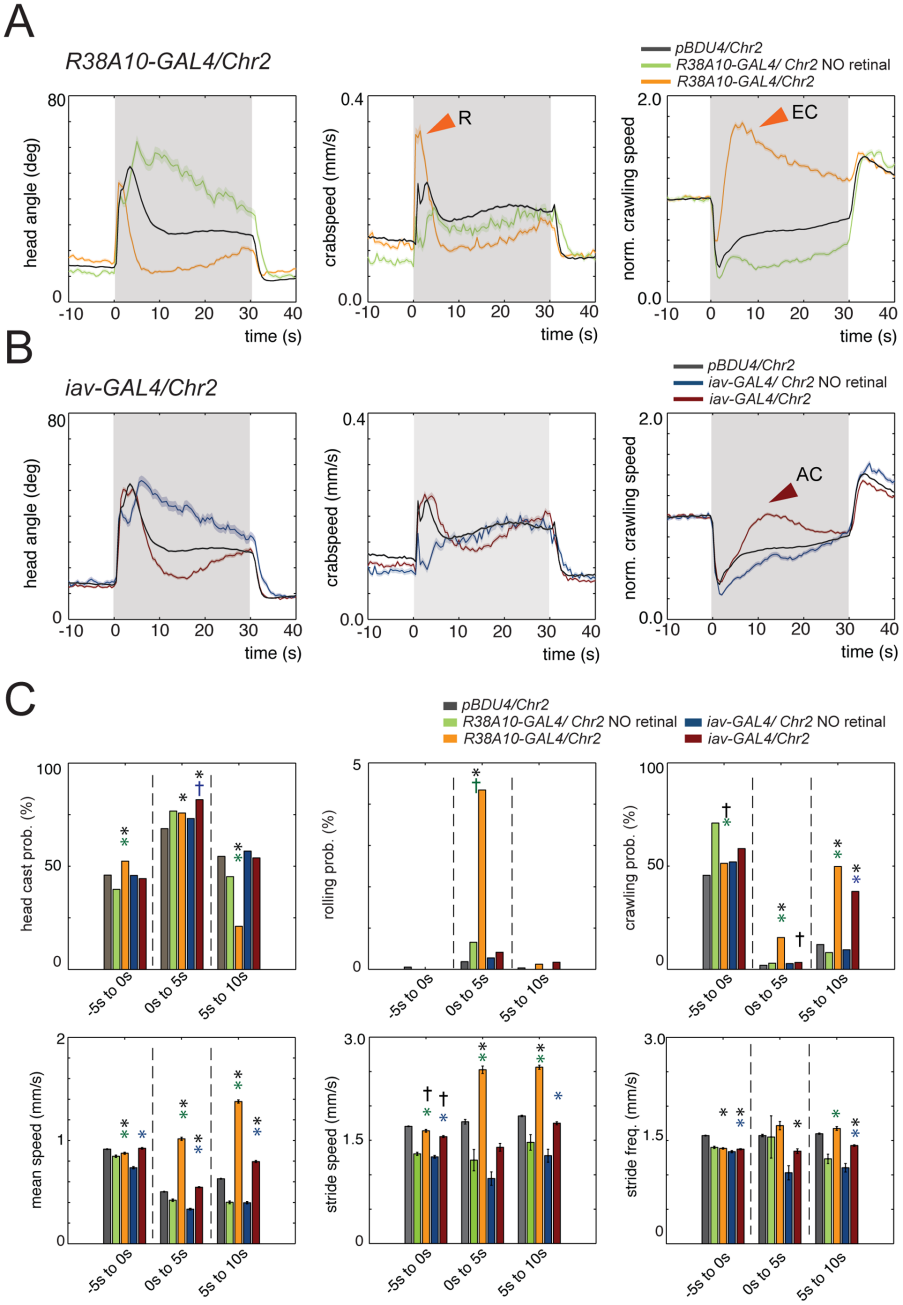
Larval reactions to optogenetic ChR2 activation of sensory neurons. (A and B) Graphs show head angle, and crabspeed and norm. crawling speed, as a function of time averaged across many animals from experiments in which larvae were presented with 30 s of continuous 470 nm light, at 25°C. Gray shading indicates the period of stimulation. Dark lines, mean value. Light lines, ± s.e.m. R, roll. EC, escape crawl. AC, avoidance crawl. Larvae with activated class IV (*R38A10>ChR2*, orange, N = 806) or ch (*iav>ChR2*, red, N = 1213) neurons are compared to no-retinal (*R38A10>ChR2* no-retinal, green, N = 305 and *iav>ChR2* no-retinal, blue, N = 361) and no-GAL4 controls (*pBDPGAL4U>ChR2*, black, N = 23305). Activation of class IV neurons evokes a peak in the crabspeed function (corresponding to the *roll*, R), followed by an increase in norm. crawling speed (corresponding to *escape crawl*, EC) compared to controls. Activation of ch neurons evokes a clear increase in norm. crawling speed during stimulation, resembling the *avoidance crawl* (AC) observed during vibration stimulation. Note also an increase in norm. crawling speed in response to 470 nm light offset in all tested lines (the off-reaction to light). (C) Bar charts show head casting, rolling and crawling probability and the absolute larval crawling speed, the maximum stride speed and stride frequency in a 5 s time window before stimulation (−5 s to 0 s) and in two consecutive 5 s time windows after stimulation (0 s to 5 s and 5 s to 10 s). Error bars indicate s.e.m. * or *, *p*<0.001.^+^or ^+^, *p*<0.01. green, blue and black, indicate comparison to *R38A10>ChR2*-no-retinal, *iav>ChR2*-no-retinal and *pBDPGAL4U>ChR2* controls, respectively. Activation of class IV neurons evokes a mild, but significant increase in rolling probability (4.3%) relative to both controls (0.6%, N = 305, *p* = 0.004468 and 0.2%, N = 23305, *p*<10^−6^ for no-retinal and no-GAL4, respectively) and a significant increase in crawling probability, mean absolute crawling speed and mean stride speed and a mild but significant increase in stride frequency (see Table S4 for details). Activation of ch neurons evokes a significant increase in head cast probability immediately following stimulation (82.3%, N = 1213) compared to both controls (73.1%, N = 361, *p* = 0.001584 and 68.2%, N = 23305, *p*<10^−6^ for non-retinal and no-GAL4, respectively) and a significant increase in crawling probability and mean absolute crawling speed relative to both controls (see Table S4 for further details).

First we characterized the reaction of the control larvae to 30 s long 470 nm light stimuli at an ambient temperature of 25°C. We found light onset induced pausing and a strong increase in head casting compared to baseline (Fig. 9A and 9B). We also found that larvae reacted strongly to the offset of light by increasing the mean speed of crawling (Fig. 9A and 9B). The observed reactions to 470 nm light onset and offset, could be mediated through both the Bolwig organ [44] or the class IV neurons, or both [45], [46].

Larvae with activated class IV neurons (*R38A10/UAS-ChR2* fed with retinal food) exhibited significantly more rolling compared to both controls, during a 30 s long blue light stimulus (Fig. 9C). This is consistent with previous studies, which showed that activation of class IV neurons was sufficient to evoke rolling using a single animal assay in a water droplet [13], [47]. However, we found that the percentage of freely crawling larvae that actually rolled in response to ChR2 activation of class IV neurons (4%, N = 802, Fig. 9C) was much lower than the percentage that rolled in response to the noxious thermal heat stimulus (ranges between 31% –36%, see Fig. 3D). In contrast we found that activation of class IV neurons in larvae that were in water induced rolling in more than 80% percent of larvae (data not shown), consistent with previous reports from single animal assays [47]. We also discovered a novel robust phenotype of activation of class IV neurons in freely crawling larvae on agar: *escape crawl* (Fig. 9A and 9C, Table S4). Interestingly, unlike the rolling component of the escape response, ChR2 activation of class IV neurons induced *escape crawling* to approximately the same level as the noxious heat stimulus.

The effect of activating ch neurons with ChR2 has previously not been described. Based on our findings that ch neurons are implicated in head casting and hunching in response to vibration and air current, and in avoidance crawling in response to vibration, we predicted that ch activation might evoke the same motor patterns. We found that activation of ch neurons (*iav-GAL4/UAS-ChR2*) did not evoke hunching, but did evoke a significant increase in head cast probability immediately following stimulation (82.3%, N = 1213, Fig. 9C, Table S4), compared to both the no-retinal (73.1%, N = 361, *p* = 0.01584), and the no-GAL4 (68.2%, N = 23305, *p<*10^−6^) controls. Activation of ch neurons also evoked a significant increase in crawling probability and mean crawling speed, compared to both controls, reminiscent of the *avoidance crawl* observed in response to vibration (Fig. 9B and 9C).

## Discussion

We developed methods that provide more than a hundredfold increase in the speed with which larval reactions to noxious, mechanical and optogenetic stimuli can be quantified compared to previously available single animal methods [26], [48]. The large sample size of our assays improves signal to noise ratio, which allows the detection of smaller behavioral effects than were detectable previously. Desired combinations and sequences of up to four stimuli can be presented at once to study multisensory integration and different forms of learning. Our system is flexible so that new stimulus modules can be added to the setup. For example, integration with described modules for automated presentation of odors could allow automated analysis of associative conditioning between somatosensory and olfactory stimuli in larvae [25]. The results from our high-throughput and high-resolution assays provide a comprehensive quantitative description of larval reactions to somatosensory stimuli and led to the identification of several novel behaviors.

### Two Different Escape Responses Evoked by Noxious Heat

We identified *escape crawl*, a novel escape behavior to noxious thermal stimuli. *Escape crawl* usually occurred after the roll and could last up to 15 s following the noxious stimulus, suggesting a short-term memory (sensitization) may be involved. The ecological relevance of this response could be that after getting away from danger (excessive heat or predator) in the most vigorous and energetically expensive way possible (rolling, 1 or 2 s with speeds up to 8 mm/s), the larva continues to escape for much longer (at 2 mm/s, but up to 15 s) to get as far away from danger, only in a less energetically costly way. Furthermore, some larvae escape crawled without rolling, and others rolled, without escape crawling, raising the interesting question about the circuit mechanisms that underlie this action-selection. The larval neural circuits may be selecting between the most vigorous but also energetically most costly and the less costly and slightly longer-term form of escape.

We found that inactivation of class IV neurons reduced both rolling and *escape crawling* to noxious heat, while their optogenetic activation evoked more *escape crawling* than rolling. Activation of class IV neurons in larvae that were immersed in water induced rolling in a higher percentage of larvae again suggesting that the effect of class IV activation on the selection of escape response could be context dependent. While larvae on agar have the choice between two escape behaviors, *escape crawling* and rolling, in water, the only choice is rolling.

We also uncovered an interesting temperature-dependence of the rolling response, where the probability of the behavior was higher at 32°C than at 25°C. Class IV neurons themselves have been shown not to respond to mild heat (32°C) [18]. Thus the synergistic effect of mild heat and noxious heat on rolling probability could be mediated through another class of sensory neuron that responds to mild heat.

### Unexpected Complexity of Larval Reactions to Mechanosensory Stimuli

We also characterized in greater detail larval reaction to vibration. We found that larvae react to vibration offset, by increasing their crawling speed (*avoidance crawl off reaction*) with respect to the baseline prior to stimulation. We described the characteristic sequence and dynamics of the larval startle, head casting (reorientation) and avoidance behaviors during continuous vibration and uncovered an interesting modulation of this behavior by temperature. During continuous vibration at 25°C, larvae crawled slower, whereas at 32°C they crawled faster, than prior to stimulation (*avoidance crawl*).

The ecological relevance of the startle, reorientation and avoidance reactions to vibration and of the interactions between the two sensory modalities (temperature and mechanosensation) remain to be elucidated. We speculate that in the larval natural habitat, vibration and sound may signal danger, for example predators such as parasitoid wasps or birds. The initial startle and reorientation response to vibration and the *avoidance off-reaction* likely offer the larva the ability to change direction of crawling in response to vibration and continue in a new direction that may be away from potential danger. Mild heat is also potentially dangerous to the animals as it could lead to desiccation. The relative balance between changing direction vs. non-directional fast avoidance during continuous vibration may be altered by temperature. Combining two potentially dangerous stimuli together could tip the decision towards a non-directional active avoidance response.

We showed that *Drosophila* larvae react to air currents and found that aspects of this reaction are different from the reaction to vibration. For example the reaction to air current offset (reducing speed and head casting more) is opposite in sign to the reaction to vibration offset (*avoidance crawl* - increasing speed and head casting less). The reaction to air current is less modulated by ambient temperature (reactions at 25°C and 32°C are not as different from each other, as the reaction to vibration). The cause of the difference in the escape strategy to air current and vibration, especially in the *off-reaction* and the ecological relevance behind this difference is unclear.

Some of the escape strategies described here are reminiscent of the strategies used during navigation in chemical or thermal gradients [25], [26], [49], [50]. During navigation larvae utilize both the non-directional klinokinesis (increase head casting and reorientation probability and decrease crawling probability if conditions are getting worse and do the opposite if conditions are getting better) and the directional klinotaxis (increase the head cast probability in the “good direction” as well as the head cast magnitude) strategies [26]. The difference between the vibration and air current off-reactions could be due to a difference in the balance between two avoidance strategies similar to the ones above. In response to air current offset (conditions got better) larvae may turn in the direction away from the direction they were in when they last sensed the stimulus (a directional klinotaxis-like response). In the case of vibration offset (conditions got better) they may simply increase their crawling probability (a non-directional klinokinesis-like response). Testing larval behavior in response to graded or directional vibration and air current in the future will help elucidate these issues.

### Larval Lateral Chordotonal Neurons Sense Vibration

We have previously shown that larval ch neurons were required for normal head casting and hunching to vibration [12]. However whether or not ch neurons respond to vibration had not been investigated. Here we show, using functional calcium imaging in ch neurons that 4 of the 5 lch neurons in each segment respond strongly to 1000 Hz vibration. This also reveals that individual larval lch neurons are functionally distinct. Whether the other lch neuron is more tuned to other frequencies, or whether it responds to other kinds of mechanosensory or other stimuli remains to be investigated. For example, a recent study has reported that v’ch responds to cooling [51], [52]. Furthermore, some ch neurons may be involved in proprioception, although we did not observe a reduction in the baseline crawling speed in larvae with inactivated ch neurons [14], [53]. In contrast, we observed an increase in the baseline crawling speed in larvae with inactivated ch neurons, relative to all three controls.

### Chordotonal Neurons are Required for Many Aspects of Larval Reactions to Mechanosensory Stimuli

We found that inactivation of all ch neurons severely impaired most aspects of the larval reaction to vibration: hunching, head casting probability and amplitude, avoidance crawling and off-reaction. Thus, ch neurons are implicated in modulating the probability of three distinct motor patterns (hunching, head casting, crawling) and the amplitude of head casting, in response to vibration. Inactivation of ch neurons also affected aspects of the reaction to air current, namely the probability of head casting and hunching and the amplitude of head casting. In the adult Johnston’s organ, distinct classes of ch neurons sense air current and vibration [43]. Whether distinct subsets of larval ch neurons sense these two stimuli remains to be elucidated. A strong residual response to air current (reduced speed throughout stimulation and a further reduction in speed, in response to air current offset), and a weak residual response to vibration (short pausing) remained in larvae with inactivated ch neurons. Thus additional somatosensory neurons may be involved in sensing air current and maybe even vibration. Alternatively the residual responses could be due to incomplete inactivation of ch neurons in our experiments.

### Optogenetic Activation of Chordotonal Neurons Increases Head Casting and Crawling Probability

Ch neurons were required for increasing the probability of head casting to air current and vibration and for increasing mean crawling speed in response to vibration onset and offset. Consistent with these inactivation results we found that optogenetic activation of ch neurons increased the probability of head casting and crawling and the mean crawling speed. Interestingly, vibration only evoked an increase in crawling speed at 32°C, and not at 25°C, but our optogenetic experiments were conducted at 25°C. A major difference between these two experiments is that 1000 Hz vibration strongly activates 4 out of the 5 lch neurons per hemisegment (Figur 6), whereas *iav-GAL4* drives expression in all ch neurons per hemisegment [51].

In general the differences observed between our experiments in which we activate specific sensory types optogenetically and experiments in which we applied stimuli could be due to several reasons. Optogenetic activation could provide weaker or stronger levels of activation compared to natural stimuli. Natural stimuli could activate only a subset of the sensory neurons within a class. Natural stimuli could activate more then one class of somatosensory neuron. In addition optogenetic activation is also accompanied with a 470 nm light stimulus. Thus all activation studies described are in the context of light, and this context is likely to modulate the behavior in various ways. Nevertheless by comparing the reaction to light alone in animals with identical genetic background to the reaction to light+optogenetic stimulation of ch or class IV neurons, we were able to identify effects of activating these neurons on behavior, that resemble aspects of the reactions to the stimuli sensed by these sensory neurons.

### Identifying Neurons and Genes Involved in Sensory-motor Transformations in the Larva

We found that each somatosensory stimulus evoked not one but several motor patterns, which tended to occur in stereotyped sequences. Distinct somatosensory stimuli appear to modulate an overlapping repertoire of motor patterns. However, they do so in distinctive modality-specific ways. This raises several interesting questions about the function of the underlying neural networks. How is the selection of each motor pattern controlled? How are the frequency, amplitude and timing of individual motor patterns controlled to generate the modality-specific dynamics? The method we have developed will allow addressing these questions in the future, using the powerful genetic toolkit for manipulating single neurons available in *Drosophila*. Our optogenetics module will allow high-throughput screens for interneurons sufficient to evoke specific motor patterns when activated with ChR2. Our modules for high-throughput presentation of somatosensory stimuli and the detailed description of wild type reactions to these stimuli provide a basis for systematic loss-of-function screens. Such screens will deliver a collection of defined neurons and genes that play key roles in larval behavior, and provide a basis for future functional imaging and electrophysiological experiments. This in turn will greatly accelerate the subsequent work on elucidating the principles of sensory integration and sensory-motor transformations, in general.

## Supporting Information

Table S1 **Summary of results from the nociception experiments.**
(CSV)

Table S2 **Summary of results from the vibration experiments.**
(CSV)

Table S3 **Summary of results from the air-current experiments.**
(CSV)

Table S4 **Summary of results from the ChR2 experiments.**
(CSV)

Movie S1 **Movie of larval reaction to noxious heat.** Movie of contours of larvae in the noxious heat assay obtained with the MWT software. Inside each contour the spine is shown as a white line divided into 10 segments. Short white lines perpendicular to the spine are used to determine the width of the animal. Green triangle marks the onset of a 1 sec long IR laser pulse that induces the noxious heat stimulus. In this experiment the black dots were painted on the back of the animals. Prior to stimulation larvae crawl and occasionally head cast and turn. In response to stimulus onset larvae strongly bend and then roll. Since the black dots are painted on the back of the animal (as shown in Figure 1B), the animals do not have a preferred direction of rolling: some roll to the left, some to the right. Following the roll animals crawl faster than prior to stimulation and we call this behavior *escape crawl*.
(AVI)

Movie S2 **Larvae roll to the let when the noxious heat stimulus is applied to the left side of the body.** Movie of contours of larvae as above. Green triangle marks the onset of a 1 sec long noxious heat stimulus. In this experiment the black dot was painted on the left side of the larvae. In response to stimulus onset larvae strongly bend and then roll. The black dots were painted on the left side of the animals (as shown in Figure 1C) and the animals roll to the left. We analyzed 17 animals with dots on the left and 15 (88%) rolled to the left, whereas 2 rolled first left then right.
(AVI)

Movie S3 **Movie from which the stills in** **Figure 2a** **were taken.** Movie of the contour of a larva as above. The black dot is painted on the back of this animal. Just prior to stimulation the larva crawled and performed a left head cast. In response a 1 sec long IR laser pulse that induced the noxious heat stimulus the larva bent and rolled and then escape crawled.
(AVI)

Movie S4 **In the absence of stimulation larvae crawl and occasionally head cast.** Movie of contours of larvae as above. In the absence of stimulation larvae crawl and occasionally head cast and change direction of crawling. When two larvae touch each other they cannot be tracked and their traces are terminated.
(AVI)

Movie S5 **Movie of the GCaMP3 imaging in ch neurons response to vibration.** A movie of Ca^2+^ signals visualized with GCaMP3 in the lch1-5 neurons of one abdominal hemisegment (A4). White circle in the top right hand corner marks stimulus onset and duration.
(AVI)

Movie S6 **Movie of larval reaction to vibration.** Movie of contours of larvae in the vibration assay obtained with the MWT software. Inside each contour the spine is shown as a white line divided into 10 segments. Green triangle marks the onset of a 30 sec long 1000 Hz 2 V vibration stimulus. White arrows point to the larvae that show clear hunching. Prior to stimulation larvae crawl and occasionally head cast and turn. In response to stimulus onset some larvae hunch (white arrows). The hunch is short and followed one or two head casts. Some larvae head cast without hunching first. Following the brief head casting phase larvae quickly resume crawling.
(AVI)

Movie S7 **Movie of larval reaction to air current.** Movie of contours of larvae in the air current assay obtained with the MWT software. Inside each contour the spine is shown as a white line divided into 10 segments. Green triangle marks the onset and duration of a 6.5 m/s air current stimulus. White arrows point to the larvae that show clear hunching. Prior to stimulation larvae crawl and occasionally head cast and turn. In response to stimulus onset some larvae hunch and others immediately head cast. Larvae perform multiple strong and long head casts and most do not resume crawling within the first 5 sec of the stimulus.
(AVI)
